# Functional Regulation of K_ATP_ Channels and Mutant Insight Into Clinical Therapeutic Strategies in Cardiovascular Diseases

**DOI:** 10.3389/fphar.2022.868401

**Published:** 2022-06-28

**Authors:** Zhicheng Wang, Weikang Bian, Yufeng Yan, Dai-Min Zhang

**Affiliations:** ^1^ Department of Cardiology, Nanjing First Hospital, Nanjing Medical University, Nanjing, China; ^2^ Department of Cardiology, Sir Run Run Hospital, Nanjing Medical University, Nanjing, China

**Keywords:** K_ATP_ channels, mitoK_ATP_ channels, myocardial ischemia, channelopathy, small active molecules, mutation

## Abstract

ATP-sensitive potassium channels (K_ATP_ channels) play pivotal roles in excitable cells and link cellular metabolism with membrane excitability. The action potential converts electricity into dynamics by ion channel-mediated ion exchange to generate systole, involved in every heartbeat. Activation of the K_ATP_ channel repolarizes the membrane potential and decreases early afterdepolarization (EAD)-mediated arrhythmias. K_ATP_ channels in cardiomyocytes have less function under physiological conditions but they open during severe and prolonged anoxia due to a reduced ATP/ADP ratio, lessening cellular excitability and thus preventing action potential generation and cell contraction. Small active molecules activate and enhance the opening of the K_ATP_ channel, which induces the repolarization of the membrane and decreases the occurrence of malignant arrhythmia. Accumulated evidence indicates that mutation of K_ATP_ channels deteriorates the regulatory roles in mutation-related diseases. However, patients with mutations in K_ATP_ channels still have no efficient treatment. Hence, in this study, we describe the role of K_ATP_ channels and subunits in angiocardiopathy, summarize the mutations of the K_ATP_ channels and the functional regulation of small active molecules in K_ATP_ channels, elucidate the potential mechanisms of mutant K_ATP_ channels and provide insight into clinical therapeutic strategies.

## Introduction

The aging of the population and improved survival after acute myocardial infarction have resulted in high morbidity and mortality, a poor clinical prognosis and high expenses due to heart failure (HF) (Savarese et al., 2022). The prevalence of HF is predicted to increase by 46% from 2012 to 2030. After several years of therapeutic exploration, the prognosis of HF remains poor, with a 5-years mortality of ≈40%–50%, and the projections suggest that the total costs for HF in 2030 will be close to $38.99 billion in the United States (Kaneko et al., 2021; Yu et al., 2021; Savarese et al., 2022). Sudden cardiac death (SCD) is the leading cause of death in HF, and malignant arrhythmia is regarded as the overriding risk within SCD (Akhtar et al., 2021; Grune et al., 2021; Mulder et al., 2021). HF involves numerous physiological and pathological processes, among which calcium (Ca^2+^) overload is a typical representative. Ca^2+^ overload destroys membranes, organelles and DNA, leading to structural and functional disruption of cells and tissues, eventually promoting cardiomyopathy, ventricular fibrillation and sudden death (Yang et al., 2021).

**FIGURE 1:**
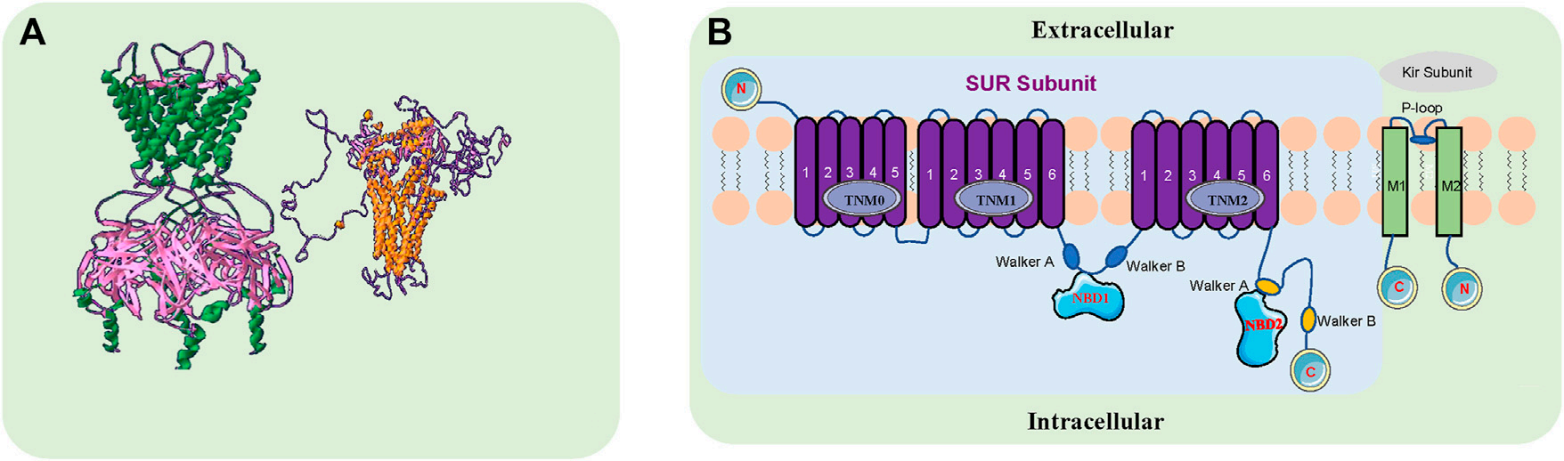
The Structure of the K_ATP_ Channel. **(A)**. K_ATP_ channels are comprised of four sulfonylurea receptors (SURx) and four K^+^ inward rectifiers (Kir6. x) that assemble to form hetero-octameric protein complexes. The green and pink sections on the left represent the side view of the Kir6. x subunit, and the gold and purple sections on the right represent the side view of the SURx subunit. FASTA comes from NCBI, designed by www.swissmodel.com, Image designed by Swiss-Pdbviewer software. **(B)**. The pore-forming subunit Kir6. x (Kir6.1 and Kir6.2) has intracellular N- and C-termini and two transmembrane segments M1 and M2, encoded by KCNJ8 and KCNJ11, respectively. The modulatory subunit SURX (SUR1, SUR2A, SUR2B) consists of three groups of transmembrane domains (TMD0, TMD1 and TMD2) and extracellular N- and intracellular C-termini, encoded by ABCC8 and ABCC9. There are two intracellular nucleotide binding folds (NBD1 and NBD2) within the SUR subunit. ABCC8 and KCNJ11 are adjacent to each other on chromosome 11p15.1, with ABCC9 and KCNJ8 on chromosome 12P12.1.

ATP-sensitive potassium channels (K_ATP_ channels) were first discovered in cardiac muscle in 1983 by Noma (Noma, 1983) and were successively found in skeletal muscle, the digestive system, urinary system, integumentary system, reproductive system, and central nervous system (Huang et al., 2019; Zhao et al., 2020) (Figure 1). Activating K_ATP_ channels shorten the action potential duration, reduce intracellular Ca^2+^ entry to suppress calcium overload, inhibit contractility, and prevent arrhythmias and cardiac insufficiency caused by calcium overload; however, completely opening K_ATP_ channels in the heart may result in complete cessation of cardiac electrical activity and contractile failure (Huang et al., 2019). Hence, K_ATP_ channels play an irreplaceable role in HF, whether from myocardial ischemia or arrhythmia.

K_ATP_ channels are widely distributed in various organs, but the assembly of their subunits varies depending upon the tissue and they may confer different functional and pharmacological properties depending on which subunits are present (Li et al., 2021; Stewart and Turner, 2021) (Table 1). K_ATP_ channels are comprised of four sulfonylurea receptors (SURx) and four K^+^ inward rectifiers (Kir6. x) that assemble to form hetero-octameric protein complexes. The pore-forming subunit Kir6. x (Kir6.1 and Kir6.2) has intracellular N- and C-termini and two transmembrane segments M1 and M2, encoded by KCNJ8 and KCNJ11, respectively. The modulatory subunit SURx (SUR1, SUR2A, SUR2B) consists of three groups of transmembrane domains (TMD0, TMD1 and TMD2) and extracellular N- and intracellular C-termini, encoded by ABCC8 and ABCC9. There are two intracellular nucleotide binding folds (NBD1 and NBD2) within the SUR subunit. ABCC8 and KCNJ11 are adjacent to each other on chromosome 11p15.1, with ABCC9 and KCNJ8 on chromosome 12P12.1.

**TABLE 1:** Distribution of K_ATP_ channels.

System	Organ/Tissue/Cell	Subunit Types	Disease Contributory
Circulatory System	Atrium		
	Ventricle	Kir6.2/SUR2B, Kir6.2/SUR1 (Zhang et al., 2013)	Atrial Fibrillation, Hypertension
	Vascular smooth muscle	Kir6.2/SUR2A (Yang et al., 2020b)	Dilated Cardiomyopathy, Myocardial Ischemia, Endothelium
	Endothelial cell	Kir6.1/SUR2B (Zhang et al., 2021a)	Dysfunction, Vasculature Atherosclerosis (Zhang et al., 2013; Wang et al., 2019b; Yang et al., 2020b; Zhang et al., 2021a)
	Capillary endothelial cell	Kir6.1/Kir6.2/SUR2B (Wang et al., 2019b)	
Respiratory System	Alveolar epithelial cells	Kir6.1/SUR2B (Leroy et al., 2006)	Pulmonary Hypertension (Leroy et al., 2006; Rieg et al., 2020)
Digestive System	Mesenteri artery	Kir6.1 (Li et al., 2020b; Jin et al., 2020)	Regulation of Blood Pressure, Excessive Atherosclerotic (Li et al., 2020b)
	Gastric smooth muscle	Kir6.1/SUR2B (Sim et al., 2002)	
	Liver	Kir6.1/Kir6.2/SUR1/SUR2A/SUR2B (Zhou et al., 2019b)	

Cardiac K_ATP_ channels provide cardioprotection against ischemia/reperfusion injury; in contrast, overexpressed cardiac K_ATP_ channels have proarrhythmic effects, which associates them with profound value for clinical applications and exploration. There are three subtypes of K_ATP_ channels found within the cardiovascular system: two widely accepted channels, mitoK_ATP_ and sarcolemma K_ATP_, and a controversial K_ATP_ channel, plasma membrane K_ATP_ (Iguchi et al., 2019; Pertiwi et al., 2019; Aziz et al., 2020; Jiang et al., 2021)_._ Different cardiac K_ATP_ channels play different roles in the cardiovascular system, which will be explained here. Recent evidence has shown that several refractory diseases are closely related to mutations in K_ATP_ channel subunits. Disease-related clinical symptoms and high medical costs will burden the patient, the family, and society. Most encouraging, some K_ATP_ channel activators and antagonists have shown good results for treating K_ATP_ channel subunit mutation-related diseases, such as Cantú syndrome, congenital hyperinsulinism (CHI), neonatal diabetes mellitus (NDM), developmental delay epilepsy and neonatal diabetes (DEND) and ABCC9-related intellectual disability myopathy Syndrome (AIMS) (Demirbilek et al., 2019; Martin et al., 2020; McClenaghan et al., 2020).

In this review, we focus on the regulatory mechanism of K_ATP_ channels during angiocardiopathy and provide insights into how mutations in K_ATP_ channelopathies lead to some incurable diseases. Furthermore, we will explore the therapeutic strategy of targeting K_ATP_ channel drugs in clinical practice.

## K_ATP_ Channels in Cardiovascular Diseases

### Mitochondrial ATP-Sensitive Potassium Channels (mitoK_ATP_ Channels)

As an independent factor associated with high mortality, acute myocardial infarction is an irreversible process characterized by glycogen depletion, margination of nuclear chromatin, mitochondrial swelling and sarcolemmal breaks. Myocardial infarct size and the duration of ischemia are the main determinants of the prognosis (Heusch, 2020). Rapidly restoring blood flow is the key to successful salvage of ischemic myocardium; however, reperfusion not only salvages ischemic myocardium from infarction but also induces an increased risk of additional complications and further cardiomyocyte death, a process called myocardial ischemia reperfusion injury (MIRI) (Griffiths et al., 2021). In myocardial ischemia, the mitochondrial matrix is damaged and extensively broken, and it dissolves the mitochondrial crest, ruptures and vacuolates the mitochondrial membrane, significantly decreases glycogen granules, increases intracellular Ca^2+^, diminishes ATP production, and induces myocardial cell apoptosis (Paggio et al., 2019; Basalay et al., 2020; Wang et al., 2020; Bai et al., 2021; Wang et al., 2021). A novel autologous mitochondrial transplantation therapy, in which respiration-competent mitochondria are isolated from autologous nonischemic tissue and transplanted into ischemic myocardium, improves the contractile function and tissue viability of the injured myocardium, proving that mitochondrial injury is the main pathogenesis of MIRI (Shin et al., 2019).

**FIGURE 2:**
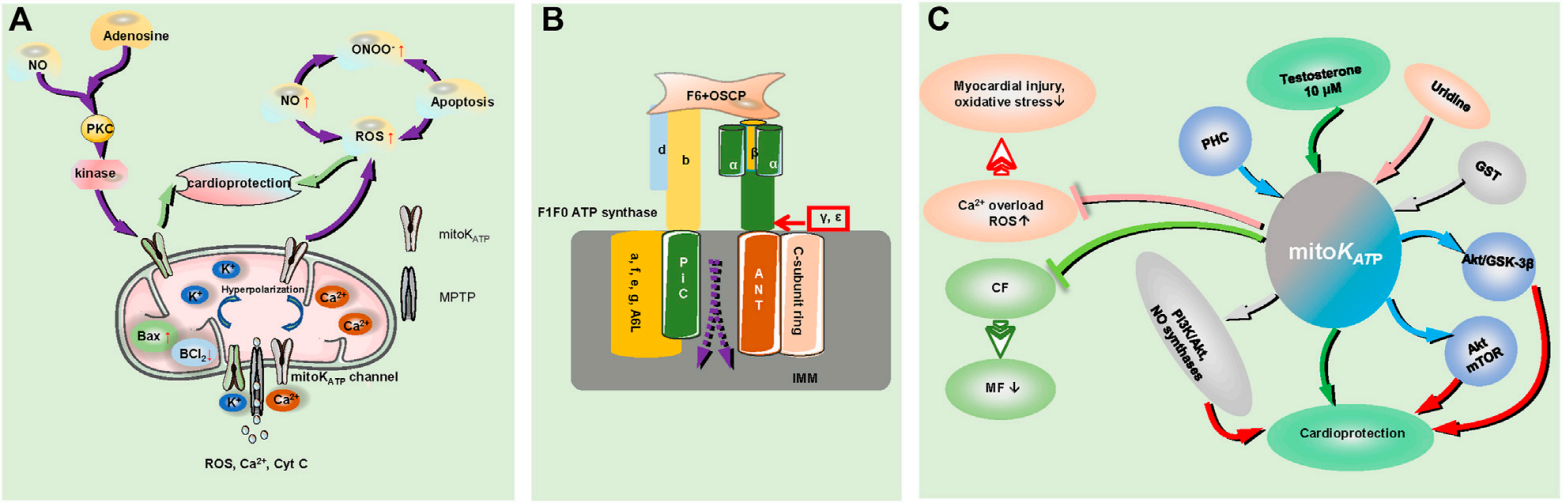
The role of mitoK_ATP_ in myocardial ischemia and the regulation of the signaling pathways involved. **(A)**. Mitochondrial injury and mitoK_ATP_ play a role in cardiac protection during the process of MIRI; MIRI increased Bax expression and decreased BCL_2_ expression, triggering a cascade involving increased NO production and leading to the superficial formation of ONOO^−^ with increased production of ROS. Activation of mitoK_ATP_ stimulates the anti-inflammatory and antioxidant effects of NO on ischemic myocardium by inhibiting the overproduction of ROS. Ischemic postadaptation activates protein kinase C and the reperfusion injury salvage kinase pathway through the intracellular concentration of adenosine and NO, and it acts on the mitoK_ATP_ channel to protect the myocardium; activation of the mitoK_ATP_ channel leads to cell hyperpolarization, resulting in reduced Ca^2+^ entry, and a reduced driving force of mitochondrial calcium uptake, prevent Ca^2+^ accumulation in the matrix and MPTP formation. The MPTP is the escape pore of ROS, Cyt C, Ca^2+^ and other signaling molecules. CF indicates cardiac fibroblasts; MF, myofibroblasts; MIRI, myocardial ischaemia reperfusion injury; and ONOO^−^, peroxynitrite. **(B)**. The structure of MPTP; F1Fo ATP synthase: The Fo subunit consists of a, e, f, g and A6 L, the F1 component consists of α and *ß* subunits, labeled in bottle green and yellow, respectively, and the γ, ε subunits. The F1 peripheral stalk is comprised of subunits b, d, F6, and OSCP. The C cyclic subunit, ANT and PiC are overlaid by IMM; the dotted line represents the MPTP signal molecule outflow position. OSCP indicates oligomycin sensitivity conferring protein; IMM, inner mitochondrial membrane; ANT, adenine nucleotide translocase; and PiC, phosphate carrier. **(C)**. mitoK_ATP_ channels prevent transdifferentiation of CF to MF to reduce MF maturation. Testosterone (10 μM) increases the opening probability of mitoK_ATP_ channels, PHC regulates the mitochondrial K_ATP_ channel, Akt/GSK-3β, and Akt/mTOR signaling pathways, GST depends on protein-kinase C and mitoK_ATP_ channel pathway activation by some typical pathways such as PI3K/Akt and NO synthases, and plays roles in cardioprotection. Uridine attenuates myocardial injury and oxidative stress by activating the mitoK_ATP_ channel to reduce excessive ROS production and prevent calcium overload. PHC indicates penehyclidine hydrochloride; GST, genistein.

mitoK_ATP_ channels are involved in a series of physiological and pathophysiological changes to mitigate cardiomyocyte injury and apoptosis. mitoK_ATP_ channels have been described as being located in the inner mitochondrial membrane and they have protective properties for ischemic myocardium; moreover, their existence has been the subject of heated debate (Bezerra Palácio et al., 2021). Recently, the molecular composition of mitoK_ATP_ was shown by Paggio et al., and they are comprised of pore-forming (MITOK, encoded by the CCDC51 gene (NCBI ID 79714)) and regulatory (MITOSUR, tissue expression correlates with ABCB8) subunits (Paggio et al., 2019) (Figure 2). The opening of mitoK_ATP_ channels promotes mitochondrial K^+^ inward flow into the deeply negative polarized matrix (mitochondrial membrane potential (*Ψ*_m_)), decreases the transmembrane potential discrepancy, depolarizes the *Ψ*_m_, reduces Ca^2+^ inward flow dynamics, inhibits Ca^2+^ inward flow, and prevents mitochondrial calcium overload, leading to mitochondrial relaxation, enhanced fatty acid oxidation, oxidative phosphorylation, respiratory function, and ATP production, thus improving myocardial cell survival (Sakamoto and Kurokawa, 2019; Jiang et al., 2021).

During the process of MIRI, the activation of mitochondrial K_ATP_ channels depolarizes the mitochondrial membrane, reduces the driving force of mitochondrial calcium uptake, and prevents Ca^2+^ accumulation in the mitochondrial matrix, thus preventing the formation of the mitochondrial permeability transition pore (MPTP) (Testai et al., 2021). Currently, the main components of MPTP are as follows: adenine nucleotide translocator (ANT) and mitochondrial phosphate carrier (PiC) on the inner mitochondrial membrane (IMM), mammalian F1Fo(F)-ATP synthase (the Fo subunit consists of a, e, f, g and A6 L; the F1 subunit *α*, *β*, and the *ε* and *γ* subunits needed for ATP synthase dimer formation, the peripheral stem consists of b, d, and the F6 subunit and oligomycin sensitivity conferring protein (OSCP)) (Kent et al., 2021) (Figure 2). MPTP is the point at which reactive oxygen species (ROS), Ca^2+^, cytochrome C (Cyt C), and other small molecule modulators escape from the mitochondrial matrix, and its opening leads to the loss of oxidative phosphorylation capacity as well as the release of pro-death mitochondrial proteins (mitochondrial swelling and membrane rupture), increased Bax expression and decreased Bcl_2_ expression, ultimately activating the apoptotic cascade in the mitochondria in ischemia–reperfusion heart tissues (Jiang et al., 2021; Kent et al., 2021; Pereira and Kowaltowski, 2021). Mitochondrial ROS spreading from the electron transport chain damages the mitochondrial DNA, which can cause mitochondrial dysfunction and affect nuclear gene expression, ion handling, and mitochondrial metabolism, finally causing the activation of an inflammatory response, apoptotic signaling, and endoplasmic reticulum stress in the cardiovascular system (Bou-Teen et al., 2021). Indeed, MIRI triggers a cascade involving increased NO production (NO function; anti-inflammatory and antioxidant effects) and leads to the superfluous formation of peroxynitrite (ONOO^−^) with increased production of ROS, which mediates the pernicious impact of NO (Liu et al., 2021a). The activation of mitoK_ATP_ during ischemia plays a role in the cardioprotective function by inhibiting the overproduction of ROS and stimulating the NO effects on anti-inflammatory and antioxidant activities in the ischemic myocardium (Liu et al., 2021a; Rameshrad et al., 2021). Ischemic postconditioning activates protein kinase C and reperfusion injury salvage kinase pathways through modulating the intracellular concentrations of adenosine and NO, ultimately acting on the mitoK_ATP_ pathway to protect the myocardium (Li et al., 2020a).

mitoK_ATP_ channels also affect other cardiac components. Within rat cardiac fibroblasts (CFs), mitoK_ATP_ channels prevent the transdifferentiation of CFs to myofibroblasts (MFs) to reduce MF maturation and antagonize cardiac pathological remodeling following simulated ischemia-reperfusion injury (Stewart and Turner, 2021). ROMK (a kidney mRNA detectable in the thick ascending limb and the distal nephron) participates in K^+^ reabsorption and secretion. An experiment performed by Irina B. Krylova et al. implicated ROMK in Ca^2+^-induced MPTP opening but did not play a role in mitoK_ATP_ activity in the mouse heart (Papanicolaou et al., 2020). Electrophysiological analysis revealed that 10 μM testosterone increased the open probability of mitoK_ATP_ channels, which offered cytoprotection against MIRI (Sakamoto and Kurokawa, 2019).

The recently discovered signal-regulated pathways involved in mitoK_ATP_ channels are as follows. Penehyclidine hydrochloride (PHC) preconditioning plays a cardioprotective role by regulating the mitochondrial K_ATP_ channel and Akt/GSK-3β and Akt/mTOR signaling pathways (Zi et al., 2020). Uridine attenuates myocardial injury and oxidative stress in MIRI, which may be mediated by activation of the mitoK_ATP_ channel, achieved by reducing excessive ROS production and preventing the appearance of calcium overload (Krylova et al., 2021). GST (genistein, a phytoestrogen) provides a cardioprotective function that depends on protein kinase C and activates mitoK_ATP_ channels via a typical pathway, such as PI3K/Akt and NO synthases (Colareda et al., 2020).

### Sarcolemma ATP-Sensitive Potassium Channels

Early evidence indicated that sarcolemma ATP-sensitive potassium (sarcK_ATP_) channels play a crucial role in ischemic preconditioning and myocardial resistance to ischemia, which close during general conditions and open in response to increased [ADP]/[ATP], linking membrane excitability to the balance of ATP production and shortening action potential (AP) duration (APD) via the efflux of K^+^ (Garrott et al., 2017; Sudhir et al., 2020). SarcK_ATP_ channels improve adaptation to physical stress and profoundly alter membrane excitability and other membrane potential-related functions, such as Ca^2+^ overload, thus helping to maintain cellular homeostasis during cardiac challenge (i.v. adenosine) (Zhang et al., 2016). The partial opening of sarcK_ATP_ channels plays a crucial role in the regional depolarization of Ψm, which can transform cellular electrical excitability and increase the propensity for reentry arrhythmogenesis (Solhjoo and O'Rourke, 2015). An unstable or oscillating Ψm can expose cardiomyocytes to ROS or result in glutathione depletion, activate sarcK_ATP_ channels and abate the cellular ATP/ADP ratio, which has been deemed to be a dominant factor in arrhythmogenesis during MIRI (Solhjoo and O'Rourke, 2015).

Increased activation of the sarcK_ATP_ channel (a role in cardioprotection) does not participate in the protection provided by ordinary cardioprotective stimulation_._ sarcK_ATP_ opening actually occurs later during metabolic inhibition (after cardioprotection), cardioprotective stimuli prolong normal mitochondrial function during ischemia, and the delay in the opening of sarcK_ATP_ channels is a consequence of the continuation of ATP production, so sarcK_ATP_ channel opening is the last defense of cardiomyocytes to preserve ATP and limit the Ca^2+^ overload during ischemia (Brennan et al., 2015). The density of sarcK_ATP_ channels under physiological conditions plays a significant role in cardioprotection; however, certain pathophysiologic circumstances give rise to a declining density of sarcK_ATP_ channels, including hyperinsulinemia and cardiac ischemia (Yang et al., 2018). The lower basal expression level of sarcK_ATP_ channels in hESCs (human embryonic stem cells)-VCMs (ventricular cardiomyocytes) (∼1/8 of adults) means they were partially activated and sufficient to cause APD shortening and accelerate AP firing; when fully activated, sarcK_ATP_ channels silenced automaticity without compromising intrinsic cellular excitability (Keung et al., 2016).

Studies on the cardiac sarcK_ATP_ channel regulatory subunit SUR2A/SUR2B are ongoing. The activation of *ß*_1_-adrenoceptors upregulates SUR2B/Kir6.2, in which SUR2B physically associates with Kir6.2 to act as a regulatory subunit in sarcK_ATP_ channels to offer cardioprotection (Jovanovic et al., 2016). With an increasing number of sarcK_ATP_ channels, increased expression of SUR2A regulates cardiac physiology and improves the adaptation to physical stress by shortening the action potential and improving cardiac Ca^2+^ homeostasis (Zhang et al., 2016).

The recently discovered signal-regulated pathways and regulatory proteins involved in sarcK_ATP_ channels are as follows. Eps15 homology domain-containing protein (EHD)-2 affects the sarcK_ATP_ channel by stabilizing sarcK_ATP_ channel-containing caveolar structures to increase its surface density, which results in a reduced rate of endocytosis. Pathophysiologically, EHD-2 mutant-activated cardiomyocytes may be cardioprotective against ischemic damage (Yang et al., 2018). In rat cardiomyocytes, the sarcK_ATP_ channel exerts a cardioprotective effect against lipopolysaccharide (LPS)-induced apoptosis and it is mediated by mitochondrial Ca^2+^ (Zhang et al., 2016). The cardioprotective effect of BNP is related to sarcK_ATP_ channel opening. Additionally, the cardioprotective effects of ANP and cANP4-23 are mediated via sarcK_ATP_ channel opening (Krylatov et al., 2021). ANP (atrial natriuretic peptide) positively regulates the function of the sarcK_ATP_ channel in adult rabbit ventricular cardiomyocytes by activating NPR-A (natriuretic peptide receptor type A), an effect mediated by intracellular signaling mechanisms that cover PKG (cGMP-dependent protein kinase), ROS, ERK (extracellular signal-regulated protein kinase)1/2, CaMK II (calcium/calmodulin-dependent protein kinase II), and RyR (ryanodine receptor)-2; meanwhile, RyR2 (activation) is feasibly situated downstream of ROS/H_2_O_2_, which process enhances the opening frequency whereas it labilizes the long closures of the channel, thereby heightening channel activity (Zhang and Lin, 2020).

## Mutation of K_ATP_ Channels

### Kir 6.1

Endothelium-expressed Kir6.1 is located on human chromosome 12p, and via elevated endothelin-1 release it controls vascular tone. Smooth muscle Kir6.1 gain-of-function mutation causes overt hypertension and hypotension; notably, autosomal dominant hypertension is related to chromosome 12p recombination, and postural hypotension is related to chromosome 12 (Li et al., 2013) (Table 2). In gain-of-function mutation Kir6.1 [GD-QR] (point mutations in two C-terminal residues of Kir6.1; Gly343Asp and Gln53Arg), lymphatic smooth muscle and vascular dysfunction are present, and lymphatic smooth muscle-specific expression subunit mutations result in profound lymphatic contractile dysfunction and lymphatic smooth muscle hyperpolarization rather than lymphatic endothelial cells (Davis et al., 2020). In a CS animal model, the Kir6.1^wt/VM^ mutation directly and/or indirectly affects the skeletal muscle through vascular dysfunction, resulting in reduced limb strength, skeletal muscle atrophy, autophagy, and myofiber connective tissue replacement (Scala et al., 2020). The S422 L mutation, a missense mutation in the KCNJ8 gene, leads to a gain-of-function Kir6.1 channel, which leads to shortened repolarization in ventricular tissue; nevertheless, it could shorten repolarization in the atrium to increase atrial fibrillation susceptibility (Delaney et al., 2012).

**TABLE 2:** Monosubunit mutation and their locus and consequence.

Mutant Subunit	Mutant Locus	Consequence
Kir 6.1	Smooth muscle	Hypertension/hypotension
	Lymphatic smooth muscle	Lymphatic contractile dysfunction lymphatic smooth muscle hyperpolarization
	Skeletal muscle	Reduced limb strength, skeletal muscle atrophy, autophagy, and myofibers connective tissue replacement
	S422L	Shortened repolarization in ventricular tissue
		Increase atrial fibrillation susceptibility
Kir 6.2	Pancreatic islet cell	Neonatal diabetes mellitus, maturity-onset diabetes of the young 13, type 2 diabetes mellitus, and even persistent hyperinsulinemic hypoglycemia of infancy
	rs5215 G/G	Vasodilation augment and shear stress reduction
	Hypertension mouse model	Heart failure and death, myocardial incommensurate remodeling
	p.V59M	Intellectual disability
SUR1	Pancreas	Neonatal diabetes
	V187D	Higher insulin secretion in hypoglycemia and make K_ATP_ channel acting pharmaceuticals out of action
	p.H1401Tfs	Clinical heterogeneity congenital hyperinsulinemia
	Unstated	Increased channel activity in MgATP/MgADP, reduced the K_ATP_ channel surface expression
SUR2A	Fs1524 and A1513T	Severely dilated hearts with impaired systolic function and arrhythmia
SUR2B	R659C	Heart disease and early repolarization syndrome
	C24S and C1455S	Prevent the detrimental effects of sulfhydration and NaHS-induced tyrosine nitration

### Kir 6.2

Approximately 38.5% of mutations in the KCNJ11 gene, which encodes Kir 6.2 and consists of a single exon containing 390 amino acids, have been identified, which is associated with clinical diseases including but not limited to neonatal diabetes mellitus, maturity-onset diabetes of the young, type 2 diabetes mellitus, and even persistent hyperinsulinemic hypoglycemia of infancy (He et al., 2021). Patients with the E227K mutation in the KCNJ11 gene typically manifest with transient neonatal diabetes, which remits spontaneously, usually within 4–60 weeks of onset; however, more than half of these patients relapse into permanent diabetes in adolescence or early adulthood (Devaraja et al., 2020). rs5215 G/G (nucleotide change; G-A, amino acid change; Val337Ile) of the KCNJ11 gene, located at 11p15.1 and encoding the Kir6.2 subunit, causes valine-isoleucine substitution in exon 1,009 (ATC-GTC), and it is associated with a gain of function of the K_ATP_ channel, leading to vasodilation augmentation and shear stress reduction, which protects humans from lower coronary microvascular dysfunction, reducing the risk of ischemic heart disease in women (Severino et al., 2020). In a hypertension mouse model, the Kir6.2 mutation led to heart failure and death, involving knockout mutation-induced myocardial incommensurate remodeling (Liu et al., 2021b). In neurons, Kir6.2 has critical roles in glucose sensing and neuronal excitability in response to metabolic demands, and the KCNJ11 p. V59 M mutation was strongly associated with intellectual disability (Moriguchi et al., 2018; Svalastoga et al., 2020).

### SUR1

SUR1 is mainly expressed in the pancreas, and its mutations may lead to neonatal diabetes by disrupting inhibitory binding/gating or enhancing nucleotide stimulation. Some SUR1 mutant models in mice did not recapitulate the human phenotype (Sachse et al., 2020; Usher et al., 2020). SUR1-mutant (a homozygous c.560T > A (V187D) mutation in exon four of the ABCC8 gene encoding the SUR1 protein) stem cell-derived islet-like clusters (SC islets) leads to increased beta-cell proliferation and mass, higher insulin secretion in hypoglycemia and makes K_ATP_ channels-acting pharmaceuticals ineffective (Lithovius et al., 2021). The homozygous p. H1401Tfs ABCC8 mutation could cause significant clinical heterogeneity congenital hyperinsulinemia, ranging from a late-onset and diazoxide-responsive mild form to an extremely early-onset severe form requiring multimodality treatment with a full-course assessment of neurodevelopment and glycometabolism (Takasawa et al., 2021). Some SUR1 mutations resulted in increased channel activity in MgATP/MgADP and drastically reduced K_ATP_ channel surface expression, which suggests that the overactive defects due to altered nucleotide sensitivities outweigh their biogenesis and surface expression defects and lead to an overall gain-of-channel-function effect and the neonatal diabetes mellitus disease phenotype (Balamurugan et al., 2019).

### SUR2A

Due to the strong difficulties and inferior feasibility of single subunit mutation research, we mainly noted several common cases herein. In individuals with idiopathic dilated cardiomyopathy, two heterozygous mutations in exon 38 of ABCC9 encode at the C-terminal domain of SUR2A, Fs1524 (a frameshift at Leu1524, which introduces four anomalous terminal residues followed by a premature stop codon) and A1513T (a missense mutation (4537G→A) causing the amino acid substitution), substantially diminishing the maximal rate of the NBD2 ATPase reaction without altering the Michaelis-Menten constant of catalysis, resulting in abnormal hydrolytic dynamics of the regulatory channel subunits, disrupting catalysis-dependent gating and impairing metabolic decoding, resulting in severely dilated hearts with impaired systolic function and arrhythmia (Bienengraeber et al., 2004).

### SUR2B

The SUR2B mutation R659C located in the secondary structure region in the L1 linker (it has the greatest *α*-helical propensity) most stably interacts with NBD1, which could cause heart disease and even lead to early repolarization syndrome, a life-threatening condition (Sooklal et al., 2018). During colonic inflammation, two specific mutations within SUR2B (C24S and C1455S) prevent the detrimental effects of sulfhydration and NaHS-induced tyrosine nitration from reducing the pore-forming subunit (Kir6.1) (Kang et al., 2015).

### Multiple Subunits Mutations of K_ATP_ Channels

Cantú syndrome (CS) is an ultrarare autosomal dominant inherited disorder caused by dominant gain-of-function mutations in both the SUR2A and Kir6.1 subunits of the K_ATP_ channel, which is also characterized by multiple cardiovascular abnormalities, including edema, pericardial effusion, pulmonary hypertension, dilated and tortuous blood vessels with decreased systemic vascular resistance, cerebrovascular defects, patent ductus arteriosus, and marked cardiac hypertrophy (Chen et al., 2019; Chihara et al., 2020; McClenaghan et al., 2020; Zhang et al., 2021a) (Table 3). CHI is a rare genetically heterogeneous disorder caused by inactivating mutations in the SUR1 and Kir6.2 subunits of the K_ATP_ channel and it is characterized by persistent hypoglycemia in infants and children, which may increase the risk of permanent brain damage (Boodhansingh et al., 2019; Rosenfeld et al., 2019; Männistö et al., 2020; Rosenfeld et al., 2021). NDM is characterized by the development of hyperglycemia within the first 6 months of life, beta-cell destruction, pancreatic hypoplasia or aplasia, impaired beta-cell function or severe insulin resistance resulting from impaired insulin secretion caused by gain-of-function mutations in KCNJ11 and/or ABCC8 subunits of the K_ATP_ channel, which can be divided into two transient diabetes mellitus (TNDM) and perma-nent diabetes mellitus (PNDM) clinical subtypes, depending on the length of the disease course (Cao et al., 2020; Dahl and Kumar, 2020; Pipatpolkai et al., 2020; Horita et al., 2021). DEND syndrome is a severe pathological condition of neonatal diabetes with developmental delay, muscle weakness, and epilepsy caused by gain-of-function mutations in Kir 6.2 and SUR1 (Dahl and Kumar, 2020; Pipatpolkai et al., 2020; Gopi et al., 2021). AIMS is characterized by delayed psychomotor development with intellectual disability, anxiety, muscle weakness and fatigability and some shared dysmorphic features caused by loss-of-function mutations in ABCC9 (SUR2A and/or SUR2B) (Smeland et al., 2019).

**TABLE 3:** Multiple subunit mutation-related disease and clinical manifestations.

Mutant Subunit	Diseases	Clinical Features
SUR2A and Kir6.1	Cantú syndrome	Edema, pericardial effusion, pulmonary hypertension, dilated and tortuous blood vessels with decreased systemic vascular resistance, and patent ductus arteriosus and cerebrovascular defects, patent ductus arteriosus, and marked cardiac hypertrophy
SUR1 and Kir6.2	Congenital hyperinsulinism	Persistent hypoglycemia in infants and children
		High risk of permanent brain damage
KCNJ11 and/or ABCC8 subunits	Neonatal diabetes	The first 6 months of life, beta-cell destruction, pancreatic hypoplasia or aplasia, impaired beta-cell function or severe insulin resistance
Kir 6.2 and SUR1	DEND syndrome	Neonatal diabetes with developmental delay, muscle weakness, and epilepsy
SUR2A and/or SUR2B	ABCC9-related intellectual disability myopathy syndrome	Intellectual disability, anxiety, muscle weakness and fatigability, and some shared dysmorphic features

## Regulation of K_ATP_ Channels by Small Active Molecules

### Hydrogen Sulfide

**FIGURE 3:**
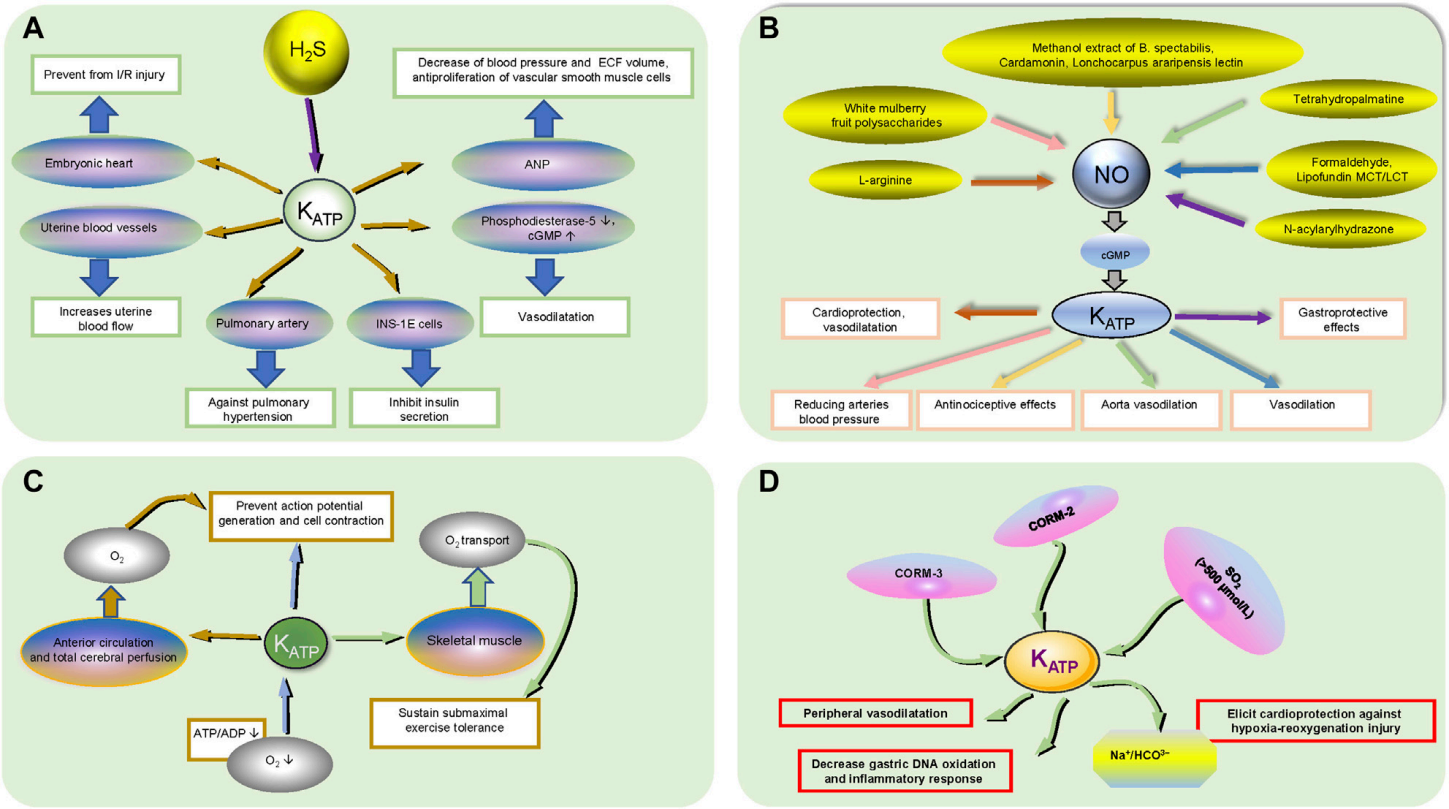
The functional regulation of active small molecules on K_ATP_ channels. **(A)** H_2_S participates in K_ATP_ channel regulation. H_2_S acts on the corresponding organ/tissues/cytochemical small molecules in the oval through K_ATP_ channels to produce physiological effects in a rectangular box. The black up and down arrows inside the ellipse represent increasing and decreasing, respectively. **(B)**. NO participates in K_ATP_ channel regulation. The relative drugs in the ellipses produce physiological effects in the rectangular boxes through the K_ATP_ channels and NO/CGMP signaling pathways, which the reaction goes in the direction of the arrows in the same color. **(C)**. O_2_ participates in K_ATP_ channel regulation. Hypoxia results in a decrease in ATP/ADP acting on K_ATP_ channels to prevent action potential generation and cell contraction; The K_ATP_ channel acts on skeletal muscle and affects O_2_ transport to sustain submaximal exercise tolerance; The K_ATP_ channel affects anterior cerebral circulation and total cerebral perfusion through O_2_ transport to prevent action potential generation and cell contraction. The black up and down arrows inside the ellipse represent increasing and decreasing, respectively. **(D)**. Other small active molecules. CORM-3 can mimic the HO-1/CO pathway, activate mitoK_ATP_ channels and elicit cardioprotection against hypoxia-reoxygenation injury by inhibiting the Na^+^/HCO_3_^−^ transporter; CORM-2 alleviates gastric lesions at the systemic level *via* K_ATP_ channels, reducing gastric DNA oxidation and inflammatory responses; SO_2_ plays a vasodilatory role through K_ATP_ channel activation in the peripheral cardiovascular system at high concentrations (>500 μmol/L).

Hydrogen sulfide (H_2_S), as a gaseous signaling molecule, has a wide range of biological functions, including vasodilatation, anti-endoplasmic reticulum stress, anti-apoptotic and anti-inflammatory functions, and it contributes to ameliorating ventricular structural remodeling and cardiac function (Li et al., 2021). In cardiac tissue, the most important enzyme for the synthesis of H_2_S is cystathionine γ-lyase (CSE), which has reduced activity in atherosclerotic patients connected with angina and atrial fibrillation (Bibli et al., 2021) (Figure 3). H_2_S has many significant bioactivities, including cytoprotective, antioxidant, anti-inflammatory, anti-apoptotic, and smooth muscle relaxing effects, in part because it acts as a K_ATP_ channel opener (Fouad et al., 2020). H_2_S partially inhibits phosphodiesterase-5 through the activation of K_ATP_ channels and increases intracellular cGMP to evoke direct vasorelaxing responses (Citi et al., 2020). H_2_S activates the K_ATP_ channel and inhibits insulin secretion in INS-1E cells (a pancreatic *ß*-cell line), but the function of hyperpolarizing the plasma membrane and closing voltage-gated Ca^2+^ channels is not mediated by the K_ATP_ channel (Lu et al., 2019; Shoji et al., 2019). H_2_S modulates K_ATP_ channel activity, promotes protective effects against pulmonary hypertension and increases uterine blood flow by antagonizing vasoconstriction (Guerra and Hurt, 2019; Roubenne et al., 2021). H_2_S protects the embryonic heart from I/R injury by opening the K_ATP_ channel rather than increasing coronary artery flow, demonstrating that H_2_S treatment of the embryonic heart is independent of the mother and the underdeveloped placenta (Hess et al., 2020). NaHS, a rapid-releasing H_2_S donor, stimulates ANP secretion via the K_ATP_ channel under hypoxic conditions, resulting in decreased blood pressure, ECF volume and antiproliferation of vascular smooth muscle cells in the cardiovascular system (Yu et al., 2019). Briefly, the interaction between H_2_S and K_ATP_ plays an irreplaceable role in cardiovascular disease.

### Nitric Oxide (NO)

The NO-cyclic guanosine monophosphate (cGMP) signaling pathway is a potential therapeutic target for heart failure, and a reduction in NO bioavailability may result in the decreased production of cGMP, which could lead to decreased protection against myocardial injury, vascular and ventricular sclerosis, fibrosis, hypertrophy, and cardiorenal syndrome (Udelson et al., 2020). K_ATP_ channels can activate the L-arginine/NO/cGMP cascade pathway to induce membrane hyperpolarization, which results in shortening the action potential and restricting Ca^2+^ entry through Ca^2+^ channels, thus contributing to cardioprotection and vasodilatation (Wang et al., 2019a; Iguchi et al., 2019). Reductions in the arterial blood pressure effect of white mulberry fruit polysaccharides, the vascular relaxation effect of tetrahydropalmatine on rat aortae, and the vasodilatory effect of formaldehyde, either partially or completely, are all mediated by the NO/cGMP/K_ATP_ pathway (Wang et al., 2019a; Zhou et al., 2019a; Zhao et al., 2019). Lipofundin MCT/LCT is involved in attenuating K_ATP_ channel-induced vasodilation by inhibiting basally released endothelial NO and/or cGMP (Lee et al., 2020). The antinociceptive effects of methanol extracts of B. spectabilis, cardamonin and Lonchocarpus araripensis lectin ether are partially or completely mediated by the NO/cGMP/K_ATP_ pathways (Assreuy et al., 2020; Ferdous et al., 2020; Pui Ping et al., 2020). The K_ATP_ channel, a gastroprotective factor, is involved in the gastroprotective effects of N-acylarylhydrazone derivatives on ethanol-induced gastric lesions in mice via the NO/cGMP pathway (da Silva Monteiro et al., 2019). Therefore, the NO/cGMP/K_ATP_ pathway is involved in a variety of organoprotective and vasodilative pharmacological processes.

### Oxygen(O_2_)

The heart operates exclusively under aerobic metabolism and three factors, heart rate, contractility, and ventricular wall tension, require myocardial mitochondria for maintaining sufficient O_2_ to sustain oxidative phosphorylation. Hypoxia causes the opening of the K_ATP_ channel due to a decline in the ATP:ADP ratio, which couples cellular metabolism to excitability to prevent action potential generation and cell contraction, ultimately leading to coronary artery smooth muscle cell hyperpolarization and the closure of voltage-dependent Ca^2+^ channels and relaxation (Yang et al., 2020a). Vascular K_ATP_ channels supporting skeletal muscle convective and diffusive O_2_ transport and oxidative phosphorylation sustain submaximal exercise tolerance; conversely, K_ATP_ channel inhibitors may exacerbate exercise intolerance in healthy rats (Colburn et al., 2020). Additionally, K_ATP_ channel activation modulates the anterior circulation and total cerebral perfusion, contributing to cerebral blood flow and oxygen delivery responses to hypoxia, maintaining a constant cerebral blood supply, avoiding disturbances in the precise regulation of cerebral perfusion and oxygen delivery, and preventing severe tissue damage and even death (Smith et al., 2020).

### Regulated by Other Factors

CORM-3, a water-soluble CO-releasing molecule that can mimic the HO-1/CO (heme oxygenase-1/carbon monoxide) pathway by liberating CO under appropriate conditions in biological systems, activates mitoK_ATP_ channels and elicits cardioprotection against hypoxia-reoxygenation injury by inhibiting a bicarbonate transporter (most likely Na^+^/HCO3^−^) during reoxygenation (Portal et al., 2019). CORM-2 increases the gastric mucosal CO content and blood carboxyhemoglobin concentration, resulting in gastroprotection, alleviation of gastric lesions, decreased gastric DNA oxidation and the inflammatory response at the systemic level, which is partly mediated by K_ATP_ channels (Li et al., 2019). Sulfur dioxide (SO_2_), a major toxic gas and environmental pollutant, plays a vasodilatory role through K_ATP_ channel activation in the peripheral cardiovascular system at high concentrations (>500 μmol/L) (Magierowska et al., 2019). The search for signaling molecular regulatory pathways related to K_ATP_ channels is still in progress.

Highly specific HCN 2 (hyperpolarization-activated, cyclic-nucleotide gated channels 2) in ventricular myocytes, an integral part of ventricular electric remodeling, and the reduced expression of its mRNA, leads to the downregulation of the K_ATP_ channel current, which is one of the partial causes of arrhythmia in diabetic rats (Hadova et al., 2021; White et al., 2021). Exogenous cholesterol eliminated the increase in SUR2, suggesting that cholesterol may regulate K_ATP_ channel expression and explain why patients with hypercholesterolemia were also able to cope with ischemic events (Geiger et al., 2021). Low-density lipoprotein (LDL)5, the most negatively charged subfraction of circulating LDL, which has been considered a novel factor for predicting coronary vascular disease and stroke, prolongs the APD and increases the current density of K_ATP_ channels, and may induce arrhythmias (Ma et al., 2020). Under 5-Hz pacing conditions, the ATP-sensitive potassium current of ZFHX3 knockdown (zinc finger homeobox three gene) cells was increased compared with that under other conditions, confirming that ZFHX3 knockdown and tachypacing are related to increased stress (Lkhagva et al., 2021). Statins increased the ADP/ATP ratio and activated K_ATP_ channels to dedifferentiate myofibroblasts, while inhibition of K_ATP_ channels weakened the role of statin-induced myofibroblast dedifferentiation (Emelyanova et al., 2019). K_ATP_ channels participate in the cardiomyocyte-specific expression of photoinduced proton pump inhibitors, hyperpolarizing the intact heart to terminate ventricular arrhythmias (Funken et al., 2019). Resistin, secreted by PVAT (the fat reserve surrounding blood vessels consisting of fat cells, immune cells, fibroblasts, and endothelial cells), did not alter K_ATP_ channel-mediated relaxation in males, while K_ATP_ channel-mediated relaxation was significantly reduced in females (Small et al., 2019).

## Clinical Therapy of Targeted K_ATP_ Channels

The functional regulation of small active molecules on K_ATP_ channels and the potential mechanisms of mutant K_ATP_ channels have been introduced in the previous content, and the relevant clinical effects and pharmacological mechanisms of some irre-placeable K_ATP_ channel openers and inhibitors will be introduced.

Nicorandil is a renowned cardioprotective drug that is characterized by opening K_ATP_ channels. It participates in the regulation of multiple signaling pathways and can be used to treat arrhythmias, chronic heart failure, stable angina, and acute coronary syndromes, including post-PCI (percutaneous coronary intervention). Nicorandil regulates coronary blood flow, protects cardiomyocytes from ischemia-reperfusion injury, alleviates endothelial dysfunction and reduces myocardial necrosis due to its K_ATP_ channel-opening effects, thereby relieving angina symptoms and limiting infarct size and subsequent severe ischemic insult (Jiang et al., 2021). A systematic review and meta-analysis demonstrated that nicorandil could effectively improve microvascular perfusion, alleviate microvascular spasms, reduce platelet aggregation, open K_ATP_ channels, reduce the excessive production of oxygen free radicals and myocardial ischemia, improve myocardial antioxidant capacity, and inhibit myocardial apoptosis and inflammatory reactions after ischemia to treat unstable angina pectoris and related microvascular complications (Zhang et al., 2021b).

Levosimendan is a calcium sensitization agent and K_ATP_ channel opener that is clinically used for the treatment of decompensated heart failure, which is characterized by inducing vasodilation of the pulmonary, coronary, and peripheral arteries and venous circulation, anti-inflammatory and antioxidant effects, and then it exerts a cardioprotective effect in various settings (Herpain et al., 2019; Efentakis et al., 2020). Levosimendan may be considered for the prevention of overt acute heart failure and cardiogenic shock due to its hemodynamic and anti-ischemia effects and its pharmacodynamic properties (Cosentino et al., 2020).

Sulfonamides, as an antibacterial drug, Marcel Janbon discovered its hypoglycemic side effects, and A. Loubatières proved that its hypoglycemic mechanism is to promote insulin secretion, so they were widely used in clinic as hypoglycemic drugs (Zhang et al., 2013). In pancreatic *ß* cells, when blood glucose concentration increases, intracellular ATP concentration increases with active glucose uptake and metabolism and inhibits K_ATP_ channels, leading to cell plasma membrane depolarization, activation of voltage-gated calcium channels, and calcium influx triggering insulin release (Yang et al., 2020b). Sulfonamides bind to K_ATP_ channel sulfonylurea receptors, inhibit the opening of K_ATP_ channel, promote the release of insulin, and reduce blood glucose and the risk of microvascular complications associated with diabetes (Wang et al., 2019b). Sulfonylureas might increase the risk of adverse cardiovascular events, due to K_ATP_ channel closure in the heart, in Neil Dhopeshwarkar et al. cohort included 268,094 glipizide users and 124,354 glimepiride users in Medicaid, they found that glimepiride (as opposed to glipizide) was associated with an elevated risk of sudden cardiac death/fatal ventricular arrhythmia, in Abdelmoneim et al. cohort included 7,441 gliclazide and 13 884 glyburide users, and they observed that statistically significant 14% higher risk of acute coronary syndrome was observed in patients taking glyburide compared with those taking gliclazide (Leroy et al., 2006; Rieg et al., 2020). In conclusion, the exploration of pancreatic specific K_ATP_ channel inhibitors can help control patients’ blood glucose, reduce microvascular complications.

At present, there is no specific pharmacotherapeutic treatment options are currently suitable for the diseases of K_ATP_ channels mutation (Rosenfeld et al., 2019). Glibenclamide inhibited K_ATP_ and slightly improved sensorimotor performance in DEND patients, but did not improve cognitive deficits caused by neuronal K_ATP_ gain-of-function expression (Jin et al., 2020). Glibenclamide directly act on SUR1, leading to the closure of K_ATP_ channel and the normal release of insulin, improving the growth imbalance, nervous system disorders and muscle strength of some PNDM children, but may cause hypoglycemia, temporary diarrhea, tooth staining, long Q-T syndrome and other adverse reactions (Cao et al., 2020). Chen et al. verified that Cantú Mutations C166S (Kir6.2) and S1020P (SUR2A) are inhibited by travoprost, betaxolol, and ritodrine, meanwhile, these compounds are not known to cause cardiac side effects or hypoglycemia (Chen et al., 2019). Scala et al. ‘s animal experiments demonstrated that glibenclamide treatment may help to reverse or avoid muscle weakness and atrophy in CS (Li et al., 2020b). In addition, in partially effective treatment regimens for patients with CHI, diazoxide opens the sarcK_ATP_ channel and inhibits insulin secretion, octreopeptide and long-acting somatostatin analogues act downstream of the K_ATP_ channel, inhibition of insulin secretion, subtotal pancreatectomy is used to reduce insulin production in focal and medically responsive non-focal cases (Rosenfeld et al., 2019). K_ATP_ openers may indeed prove beneficial in some AIMS patients (Bibli et al., 2021). K_ATP_ channel activator tifenazoxide, VU0071063 can be unlocked by opening the K_ATP_ channel, providing CHI patients with a new pharmacological option for CHI therapy to maintain normal blood glucose and reduce drug side effects and postoperative complications (Sim et al., 2002). Interestingly, CRISPR-based genome editing techniques were found to detect changes in ABCC8 and SUR1 expression levels in type 2 diabetes, suggesting that gene editing could be useful in diagnosing and treating K_ATP_ channel mutations in the future (Zhou et al., 2019b).

## Conclusion

At present, no specific pharmacotherapeutic treatment options are currently suitable for CS, but glibenclamide can partially reverse the vascular symptoms of CS by inhibiting the overactivity of the K_ATP_ channel (McClenaghan et al., 2020; Laimon et al., 2021). Sulfonylureas can inhibit the effect of ABCC8 and KCNJ11 activation mutations that prevent the closure of K_ATP_ channels leading to insulin deficiency, reversing a condition that has historically been treated only with insulin (Laimon et al., 2021). The early ascertainment of a genetic diagnosis help us find the underlying cause which is the optimal treatment of the diseases of mutations in K_ATP_ channels. These facts support the hypothesis that the study of K_ATP_ channels may improve the prognosis, alleviate pain, and reduce the economic burden on patients. In the short run, Patients with cardiovascular disease and refractory K_ATP_ channel subunit mutations will have more treatment options, more promising outcomes, and acceptable medical costs.

## Glossary

ADP: adenosine diphosphate
AIMS: ABCC9-related intellectual disability myopathy Syndrome
ANP: atrial natriuretic peptide
ANT: adenine nucleotide translocator
AP: action potential
APD: action potential duration
ATP: adenosine triphosphate
BCL2: B-cell lymphoma-2
CaMK: calcium/calmodulin-dependent protein kinase
CF: cardiac fibroblasts
cGMP: cyclic guanosine monophosphate
CHI: congenital hyperinsulinism
CO: carbon monoxide
CORM-3: CO-releasing molecule-3
CS: cantú syndrome
CSE: cystathionine *γ*-lyase
Cyt C: cytochrome C
DEND syndrome: developmental delay epilepsy and neonatal diabetes syndrome
EAD: early afterdepolarization
EHD-2: Eps15 homology domain-containing protein-2
ERK: extracellular signal-regulated protein kinase
GST: genistein
H_2_S: hydrogen sulfide
HCN: hyperpolarization-activated cyclic-nucleotide gated channels
hESC: human embryonic stem cell
HF: heart failure
HO-1/CO: heme oxygenase-1/carbon monoxide
IMM: inner mitochondrial membrane
INS-1E cells: a pancreatic *ß* cell line
K^+^: potassium
KATP channels: ATP-sensitive potassium channels;
Kir: K^+^ inward rectifiers
LDL: low-density lipoprotein
LPS: lipopolysaccharide
MF: myofibroblasts
MIRI: myocardial ischaemia reperfusion injury
mitoKATP: mitochondrial ATP-sensitive potassium
MPTP: permeability transition pore
NBF: nucleotide binding folds
NDM: neonatal diabetes mellitus
NO: nitric oxide
NPR-A: natriuretic peptide receptor type A
O_2_: oxygen
ONOO-: peroxynitrite
PCI: percutaneous coronary intervention
PHC: penehyclidine hydrochloride
PNDM: permanent neonatal diabetes mellitus
PiC: phosphate carrier
PKG: cGMP-dependent protein kinase
PLN: phospholamban
ROS: reactive oxygen species
RyR: ryanodine receptor
sarcKATP: sarcolemma ATP-sensitive potassium
SCD: sudden cardiac death
SO_2_: sulfur dioxide
SUR: sulfonylurea receptors
TMD: transmembrane domains
TNDM: transient neonatal diabetes mellitus
VCMs: ventricular cardiomyocytes
ZFHX3: zinc finger homeobox three gene

## References

[B1] Akhtar M. M. Lorenzini M. Pavlou M. Ochoa J. P. O'Mahony C. Restrepo-Cordoba M. A. (2021). Association of Left Ventricular Systolic Dysfunction Among Carriers of Truncating Variants in Filamin C with Frequent Ventricular Arrhythmia and End-Stage Heart Failure. JAMA Cardiol. 6 (8), 891–901. 10.1001/jamacardio.2021.1106 33978673 PMC8117057

[B2] Assreuy A. M. S. Amorim R. M. F. Martins S. L. de Queiroz Martins M. G. Cajazeiras J. B. da Silva M. T. L. (2020). Antinociceptive Effect of Lonchocarpus Araripensis Lectin: Activation of L-arginine/NO/cGMP/K+ATP Signaling Pathway. Inflammopharmacology 28 (6), 1623–1631. 10.1007/s10787-020-00729-z 32572724

[B3] Aziz Q. Chen J. Moyes A. J. Li Y. Anderson N. A. Ang R. (2020). Vascular KATP Channels Protect from Cardiac Dysfunction and Preserve Cardiac Metabolism during Endotoxemia. J. Mol. Med. Berl. 98 (8), 1149–1160. 10.1007/s00109-020-01946-3 32632751 PMC7399691

[B4] Bai S. Wang X. Wu H. Chen T. Li X. Zhang L. (2021). Cardioprotective Effect of Anisodamine against Ischemia/reperfusion Injury through the Mitochondrial ATP-Sensitive Potassium Channel. Eur. J. Pharmacol. 901, 174095. 10.1016/j.ejphar.2021.174095 33862063

[B5] Balamurugan K. Kavitha B. Yang Z. Mohan V. Radha V. Shyng S. L. (2019). Functional Characterization of Activating Mutations in the Sulfonylurea Receptor 1 (ABCC8) Causing Neonatal Diabetes Mellitus in Asian Indian Children. Pediatr. Diabetes 20 (4), 397–407. 10.1111/pedi.12843 30861254 PMC11423867

[B6] Basalay M. V. Yellon D. M. Davidson S. M. (2020). Targeting Myocardial Ischaemic Injury in the Absence of Reperfusion. Basic Res. Cardiol. 115 (6), 63. 10.1007/s00395-020-00825-9 33057804 PMC7560937

[B7] Bezerra Palácio P. Brito Lucas A. M. Varlla de Lacerda Alexandre J. Oliveira Cunha P. L. Ponte Viana Y. I. Albuquerque A. C. (2021). Pharmacological and Molecular Docking Studies Reveal that Glibenclamide Competitively Inhibits Diazoxide-Induced Mitochondrial ATP-Sensitive Potassium Channel Activation and Pharmacological Preconditioning. Eur. J. Pharmacol. 908, 174379. 10.1016/j.ejphar.2021.174379 34324857

[B8] Bibli S. I. Hu J. Looso M. Weigert A. Ratiu C. Wittig J. (2021). Mapping the Endothelial Cell S-Sulfhydrome Highlights the Crucial Role of Integrin Sulfhydration in Vascular Function. Circulation 143 (9), 935–948. 10.1161/CIRCULATIONAHA.120.051877 33307764

[B9] Bienengraeber M. Olson T. M. Selivanov V. A. Kathmann E. C. O'Cochlain F. Gao F. (2004). ABCC9 Mutations Identified in Human Dilated Cardiomyopathy Disrupt Catalytic KATP Channel Gating. Nat. Genet. 36 (4), 382–387. 10.1038/ng1329 15034580 PMC1995438

[B10] Boodhansingh K. E. Kandasamy B. Mitteer L. Givler S. De Leon D. D. Shyng S. L. (2019). Novel Dominant KATP Channel Mutations in Infants with Congenital Hyperinsulinism: Validation by *In Vitro* Expression Studies and *In Vivo* Carrier Phenotyping. Am. J. Med. Genet. A 179 (11), 2214–2227. 10.1002/ajmg.a.61335 31464105 PMC6852436

[B11] Bou-Teen D. Kaludercic N. Weissman D. Turan B. Maack C. Di Lisa F. (2021). Mitochondrial ROS and Mitochondria-Targeted Antioxidants in the Aged Heart. Free Radic. Biol. Med. 167, 109–124. 10.1016/j.freeradbiomed.2021.02.043 33716106

[B12] Brennan S. Jackson R. Patel M. Sims M. W. Hudman D. Norman R. I. (2015). Early Opening of Sarcolemmal ATP-Sensitive Potassium Channels Is Not a Key Step in PKC-Mediated Cardioprotection. J. Mol. Cell Cardiol. 79, 42–53. 10.1016/j.yjmcc.2014.10.016 25450614

[B13] Cao L. He Y. Huang Q. Zhang Y. Deng P. Du W. (2020). Clinical Features and Partial Proportional Molecular Genetics in Neonatal Diabetes Mellitus: a Retrospective Analysis in Southwestern China. Endocrine 69 (1), 53–62. 10.1007/s12020-020-02279-4 32279225

[B14] Chen X. Garon A. Wieder M. Houtman M. J. C. Zangerl-Plessl E. M. Langer T. (2019). Computational Identification of Novel Kir6 Channel Inhibitors. Front. Pharmacol. 10, 549. 10.3389/fphar.2019.00549 31178728 PMC6543810

[B15] Chihara M. Asahina A. Itoh M. (2020). A Novel Mutation in the KCNJ8 Gene Encoding the Kir6.1 Subunit of an ATP-Sensitive Potassium Channel in a Japanese Patient with Cantú Syndrome. J. Eur. Acad. Dermatol Venereol. 34 (9), e476–e478. 10.1111/jdv.16384 32215968

[B16] Citi V. Martelli A. Bucci M. Piragine E. Testai L. Vellecco V. (2020). Searching for Novel Hydrogen Sulfide Donors: The Vascular Effects of Two Thiourea Derivatives. Pharmacol. Res. 159, 105039. 10.1016/j.phrs.2020.105039 32565313

[B17] Colareda G. A. Ragone M. I. Bonazzola P. Consolini A. E. (2020). The mKATP Channels and Protein-Kinase C Are Involved in the Cardioprotective Effects of Genistein on Estrogen-Deficient Rat Hearts Exposed to Ischemia/Reperfusion: Energetic Study. J. Cardiovasc Pharmacol. 75 (5), 460–474. 10.1097/FJC.0000000000000816 32195757

[B18] Colburn T. D. Weber R. E. Hageman K. S. Caldwell J. T. Schulze K. M. Ade C. J. (2020). Vascular ATP-Sensitive K+ Channels Support Maximal Aerobic Capacity and Critical Speed via Convective and Diffusive O2 Transport. J. Physiol. 598 (21), 4843–4858. 10.1113/JP280232 32798233 PMC7874302

[B19] Cosentino N. Niccoli G. Fracassi F. Rebuzzi A. Agostoni P. Marenzi G. (2020). Rationale, Experimental Data, and Emerging Clinical Evidence on Early and Preventive Use of Levosimendan in Patients with Ventricular Dysfunction. Eur. Heart J. Cardiovasc Pharmacother. 6 (5), 310–316. 10.1093/ehjcvp/pvz065 31688906

[B20] da Silva Monteiro C. E. Franco Á. X. Sousa J. A. O. Matos V. E. A. de Souza E. P. Fraga C. A. M. (2019). Gastroprotective Effects of N-Acylarylhydrazone Derivatives on Ethanol-Induced Gastric Lesions in Mice Are Dependent on the NO/cGMP/KATP Pathway. Biochem. Pharmacol. 169, 113629. 10.1016/j.bcp.2019.113629 31491412

[B21] Dahl A. Kumar S. (2020). Recent Advances in Neonatal Diabetes. Diabetes Metab. Syndr. Obes. 13, 355–364. 10.2147/DMSO.S198932 32104032 PMC7024796

[B22] Davis M. J. Kim H. J. Zawieja S. D. Castorena-Gonzalez J. A. Gui P. Li M. (2020). Kir6.1-dependent KATP Channels in Lymphatic Smooth Muscle and Vessel Dysfunction in Mice with Kir6.1 Gain-Of-Function. J. Physiol. 598 (15), 3107–3127. 10.1113/JP279612 32372450 PMC7641979

[B23] Delaney J. T. Muhammad R. Blair M. A. Kor K. Fish F. A. Roden D. M. (2012). A KCNJ8 Mutation Associated with Early Repolarization and Atrial Fibrillation. Europace 14 (10), 1428–1432. 10.1093/europace/eus150 22562657 PMC3458578

[B24] Demirbilek H. Galcheva S. Vuralli D. Al-Khawaga S. Hussain K. (2019). Ion Transporters, Channelopathies, and Glucose Disorders. Int. J. Mol. Sci. 20 (10), 2590. 10.3390/ijms20102590 PMC6566632 31137773

[B25] Devaraja J. Elder C. Scott A. (2020). Non Classic Presentations of a Genetic Mutation Typically Associated with Transient Neonatal Diabetes. Endocrinol. Diabetes Metab. Case Rep. 2020, 2020. 10.1530/EDM-19-0125 PMC7077548 32101525

[B26] Efentakis P. Varela A. Chavdoula E. Sigala F. Sanoudou D. Tenta R. (2020). Levosimendan Prevents Doxorubicin-Induced Cardiotoxicity in Time- and Dose-dependent Manner: Implications for Inotropy. Cardiovasc Res. 116 (3), 576–591. 10.1093/cvr/cvz163 31228183

[B27] Emelyanova L. Sra A. Schmuck E. G. Raval A. N. Downey F. X. Jahangir A. (2019). Impact of Statins on Cellular Respiration and De-differentiation of Myofibroblasts in Human Failing Hearts. Esc. Heart Fail 6 (5), 1027–1040. 10.1002/ehf2.12509 31520523 PMC6816080

[B28] Ferdous A. Janta R. A. Arpa R. N. Afroze M. Khan M. Moniruzzaman M. (2020). The Leaves of Bougainvillea Spectabilis Suppressed Inflammation and Nociception *In Vivo* through the Modulation of Glutamatergic, cGMP, and ATP-Sensitive K+ Channel Pathways. J. Ethnopharmacol. 261, 113148. 10.1016/j.jep.2020.113148 32687959

[B29] Fouad A. A. Hafez H. M. Hamouda A. (2020). Hydrogen Sulfide Modulates IL-6/STAT3 Pathway and Inhibits Oxidative Stress, Inflammation, and Apoptosis in Rat Model of Methotrexate Hepatotoxicity. Hum. Exp. Toxicol. 39 (1), 77–85. 10.1177/0960327119877437 31542963

[B30] Funken M. Malan D. Sasse P. Bruegmann T. (2019). Optogenetic Hyperpolarization of Cardiomyocytes Terminates Ventricular Arrhythmia. Front. Physiol. 10, 498. 10.3389/fphys.2019.00498 31105593 PMC6491897

[B31] Garrott K. Kuzmiak-Glancy S. Wengrowski A. Zhang H. Rogers J. Kay M. W. (2017). KATP Channel Inhibition Blunts Electromechanical Decline during Hypoxia in Left Ventricular Working Rabbit Hearts. J. Physiol. 595 (12), 3799–3813. 10.1113/JP273873 28177123 PMC5471424

[B32] Geiger R. Fatima N. Schooley J. F. Jr. Smyth J. T. Haigney M. C. Flagg T. P. (2021). Novel Cholesterol-dependent Regulation of Cardiac KATP Subunit Expression Revealed Using Histone Deacetylase Inhibitors. Physiol. Rep. 8 (24), e14675. 10.14814/phy2.14675 33356020 PMC7757372

[B33] Gopi S. Kavitha B. Kanthimathi S. Kannan A. Kumar R. Joshi R. (2021). Genotype-phenotype Correlation of KATP Channel Gene Defects Causing Permanent Neonatal Diabetes in Indian Patients. Pediatr. Diabetes 22 (1), 82–92. 10.1111/pedi.13109 32893419

[B34] Griffiths K. Lee J. J. Frenneaux M. P. Feelisch M. Madhani M. (2021). Nitrite and Myocardial Ischaemia Reperfusion Injury. Where Are We Now? Pharmacol. Ther. 223, 107819. 10.1016/j.pharmthera.2021.107819 33600852

[B35] Grune J. Yamazoe M. Nahrendorf M. (2021). Electroimmunology and Cardiac Arrhythmia. Nat. Rev. Cardiol. 18 (8), 547–564. 10.1038/s41569-021-00520-9 33654273 PMC9703448

[B36] Guerra D. D. Hurt K. J. (2019). Gasotransmitters in Pregnancy: from Conception to Uterine Involution. Biol. Reprod. 101 (1), 4–25. 10.1093/biolre/ioz038 30848786 PMC6614580

[B37] Hadova K. Kralova E. Doka G. Bies Pivackova L. Kmecova Z. Krenek P. (2021). Isolated Downregulation of HCN2 in Ventricles of Rats with Streptozotocin-Induced Diabetic Cardiomyopathy. BMC Cardiovasc Disord. 21 (1), 118. 10.1186/s12872-021-01929-3 33653265 PMC7927235

[B38] He B. Li X. Zhou Z. (2021). Continuous Spectrum of Glucose Dysmetabolism Due to the KCNJ11 Gene Mutation-Case Reports and Review of the Literature. J. Diabetes 13 (1), 19–32. 10.1111/1753-0407.13114 32935446

[B39] Herpain A. Bouchez S. Girardis M. Guarracino F. Knotzer J. Levy B. (2019). Use of Levosimendan in Intensive Care Unit Settings: An Opinion Paper. J. Cardiovasc Pharmacol. 73 (1), 3–14. 10.1097/FJC.0000000000000636 30489437 PMC6319595

[B40] Hess R. M. Niu Y. Garrud T. A. C. Botting K. J. Ford S. G. Giussani D. A. (2020). Embryonic Cardioprotection by Hydrogen Sulphide: Studies of Isolated Cardiac Function and Ischaemia-Reperfusion Injury in the Chicken Embryo. J. Physiol. 598 (19), 4197–4208. 10.1113/JP279978 32705691

[B41] Heusch G. (2020). Myocardial Ischaemia-Reperfusion Injury and Cardioprotection in Perspective. Nat. Rev. Cardiol. 17 (12), 773–789. 10.1038/s41569-020-0403-y 32620851

[B42] Horita S. Ono T. Gonzalez-Resines S. Ono Y. Yamachi M. Zhao S. (2021). Structure Based Analysis of KATP Channel with a DEND Syndrome Mutation in Murine Skeletal Muscle. Sci. Rep. 11 (1), 6668. 10.1038/s41598-021-86121-5 33758250 PMC7988048

[B43] Huang Y. Hu D. Huang C. Nichols C. G. (2019). Genetic Discovery of ATP-Sensitive K+ Channels in Cardiovascular Diseases. Circ. Arrhythm. Electrophysiol. 12 (5), e007322. 10.1161/CIRCEP.119.007322 31030551 PMC6494091

[B44] Iguchi K. Saotome M. Yamashita K. Hasan P. Sasaki M. Maekawa Y. (2019). Pinacidil, a KATP Channel Opener, Stimulates Cardiac Na+/Ca2+ Exchanger Function through the NO/cGMP/PKG Signaling Pathway in guinea Pig Cardiac Ventricular Myocytes. Schmiedeb. Arch. Pharmacol. 392 (8), 949–959. 10.1007/s00210-019-01642-1 30919008

[B45] Jiang X. Wu D. Jiang Z. Ling W. Qian G. (2021). Protective Effect of Nicorandil on Cardiac Microvascular Injury: Role of Mitochondrial Integrity. Oxid. Med. Cell Longev. 2021, 4665632. 10.1155/2021/4665632 34285763 PMC8275446

[B46] Jin X. Wu Y. Cui N. Jiang C. Li S. S. (2020). Methylglyoxal-induced miR-223 Suppresses Rat Vascular KATP Channel Activity by Downregulating Kir6.1 mRNA in Carbonyl Stress. Vasc. Pharmacol. 128-129, 106666. 10.1016/j.vph.2020.106666 32151743

[B47] Jovanovic S. Ballantyne T. Du Q. Blagojević M. Jovanović A. (2016). Phenylephrine Preconditioning in Embryonic Heart H9c2 Cells Is Mediated by Up-Regulation of SUR2B/Kir6.2: A First Evidence for Functional Role of SUR2B in Sarcolemmal K_ATP_ Channels and Cardioprotection. Int. J. Biochem. Cell Biol. 70, 23–28. 10.1016/j.biocel.2015.10.029 26556311 PMC4711337

[B48] Kaneko H. Yano Y. Itoh H. Morita K. Kiriyama H. Kamon T. (2021). Association of Blood Pressure Classification Using the 2017 American College of Cardiology/American Heart Association Blood Pressure Guideline with Risk of Heart Failure and Atrial Fibrillation. Circulation 143 (23), 2244–2253. 10.1161/CIRCULATIONAHA.120.052624 33886370

[B49] Kang M. Hashimoto A. Gade A. Akbarali H. I. (2015). Interaction between Hydrogen Sulfide-Induced Sulfhydration and Tyrosine Nitration in the KATP Channel Complex. Am. J. Physiol. Gastrointest. Liver Physiol. 308 (6), G532–G539. 10.1152/ajpgi.00281.2014 25552582 PMC4360042

[B50] Kent A. C. El Baradie K. B. Y. Hamrick M. W. (2021). Targeting the Mitochondrial Permeability Transition Pore to Prevent Age-Associated Cell Damage and Neurodegeneration. Oxid. Med. Cell Longev. 2021, 6626484. 10.1155/2021/6626484 33574977 PMC7861926

[B51] Keung W. Ren L. Sen Li L. Wong A. O. Chopra A. Kong C. W. (2016). Non-cell Autonomous Cues for Enhanced Functionality of Human Embryonic Stem Cell-Derived Cardiomyocytes via Maturation of Sarcolemmal and Mitochondrial KATP Channels. Sci. Rep. 6, 34154. 10.1038/srep34154 27677332 PMC5039730

[B52] Krylatov A. V. Tsibulnikov S. Y. Mukhomedzyanov A. V. Boshchenko A. A. Goldberg V. E. Jaggi A. S. (2021). The Role of Natriuretic Peptides in the Regulation of Cardiac Tolerance to Ischemia/Reperfusion and Postinfarction Heart Remodeling. J. Cardiovasc Pharmacol. Ther. 26 (2), 131–148. 10.1177/1074248420952243 32840121

[B53] Krylova I. B. Selina E. N. Bulion V. V. Rodionova O. M. Evdokimova N. R. Belosludtseva N. V. (2021). Uridine Treatment Prevents Myocardial Injury in Rat Models of Acute Ischemia and Ischemia/reperfusion by Activating the Mitochondrial ATP-dependent Potassium Channel. Sci. Rep. 11 (1), 16999. 10.1038/s41598-021-96562-7 34417540 PMC8379228

[B54] Laimon W. El-Ziny M. El-Hawary A. Elsharkawy A. Salem N. A. Aboelenin H. M. (2021). Genetic and Clinical Heterogeneity of Permanent Neonatal Diabetes Mellitus: a Single Tertiary Centre Experience. Acta Diabetol. 58 (12), 1689–1700. 10.1007/s00592-021-01788-6 34426871

[B55] Lee S. H. Kang D. Ok S. H. Kim J. Y. Bae S. I. Hwang Y. (2020). Lipofundin MCT/LCT Inhibits Levcromakalim-Induced Vasodilation by Inhibiting Endothelial Nitric Oxide Release. Int. J. Mol. Sci. 21 (5), 1763. 10.3390/ijms21051763 PMC7084418 32143531

[B56] Leroy C. Privé A. Bourret J. C. Berthiaume Y. Ferraro P. Brochiero E. (2006). Regulation of ENaC and CFTR Expression with K+ Channel Modulators and Effect on Fluid Absorption across Alveolar Epithelial Cells. Am. J. Physiol. Lung Cell Mol. Physiol. 291 (6), L1207–L1219. 10.1152/ajplung.00376.2005 16891388

[B57] Li A. Knutsen R. H. Zhang H. Osei-Owusu P. Moreno-Dominguez A. Harter T. M. (2013). Hypotension Due to Kir6.1 Gain-Of-Function in Vascular Smooth Muscle. J. Am. Heart Assoc. 2 (4), e000365. 10.1161/JAHA.113.000365 23974906 PMC3828800

[B58] Li B. Gao M. X. Yang W. L. Chai C. Zhang D. X. Cai H. Y. (2019). Inhibitory Effects of Sulfur Dioxide within the Nucleus Tractus Solitarii of Rats: Involvement of Calcium Ion Channels, Adenine Nucleoside Triphosphate-Sensitive Potassium Channels, and the Nitric Oxide/cyclic Guanine Trinucleotide Phosphate Pathway. Neuroreport 30 (13), 914–920. 10.1097/WNR.0000000000001304 31373972 PMC6686961

[B59] Li J. Zhou W. Chen W. Wang H. Zhang Y. Yu T. (2020). Mechanism of the Hypoxia Inducible Factor 1/hypoxic Response Element Pathway in Rat Myocardial Ischemia/diazoxide Post-conditioning. Mol. Med. Rep. 21 (3), 1527–1536. 10.3892/mmr.2020.10966 32016463 PMC7003038

[B60] Li Y. Aziz Q. Anderson N. Ojake L. Tinker A. (2020). Endothelial ATP-Sensitive Potassium Channel Protects against the Development of Hypertension and Atherosclerosis. Hypertension 76 (3), 776–784. 10.1161/HYPERTENSIONAHA.120.15355 32654556 PMC7418932

[B61] Li Y. Feng Y. Liu L. Li X. Li X. Y. Sun X. (2021). The Baroreflex Afferent Pathway Plays a Critical Role in H2S-Mediated Autonomic Control of Blood Pressure Regulation under Physiological and Hypertensive Conditions. Acta Pharmacol. Sin. 42 (6), 898–908. 10.1038/s41401-020-00549-5 33154555 PMC8149652

[B62] Lithovius V. Saarimäki-Vire J. Balboa D. Ibrahim H. Montaser H. Barsby T. (2021). SUR1-mutant iPS Cell-Derived Islets Recapitulate the Pathophysiology of Congenital Hyperinsulinism. Diabetologia 64 (3), 630–640. 10.1007/s00125-020-05346-7 33404684

[B63] Liu C. Lai Y. Pei J. Huang H. Zhan J. Ying S. (2021). Clinical and Genetic Analysis of KATP Variants with Heart Failure Risk in Patients with Decreased Serum ApoA-I Levels. J. Clin. Endocrinol. Metab. 106 (8), 2264–2278. 10.1210/clinem/dgab336 33982099

[B64] Liu Y. Song Y. Li S. Mo L. (2021). Cardioprotective Effect of Quercetin against Ischemia/Reperfusion Injury Is Mediated through NO System and Mitochondrial K-ATP Channels. Cell J. 23 (2), 184–190. 10.22074/cellj.2021.7183 34096219 PMC8181321

[B65] Lkhagva B. Lin Y. K. Chen Y. C. Cheng W. L. Higa S. Kao Y. H. (2021). ZFHX3 Knockdown Dysregulates Mitochondrial Adaptations to Tachypacing in Atrial Myocytes through Enhanced Oxidative Stress and Calcium Overload. Acta Physiol. (Oxf) 231 (4), e13604. 10.1111/apha.13604 33332716

[B66] Lu A. Chu C. Mulvihill E. Wang R. Liang W. (2019). ATP-sensitive K+ Channels and Mitochondrial Permeability Transition Pore Mediate Effects of Hydrogen Sulfide on Cytosolic Ca2+ Homeostasis and Insulin Secretion in β-cells. Pflugers Arch. 471 (11-12), 1551–1564. 10.1007/s00424-019-02325-9 31713764

[B67] Ma Y. Cheng N. Sun J. Lu J. X. Abbasi S. Wu G. (2020). Atherogenic L5 LDL Induces Cardiomyocyte Apoptosis and Inhibits KATP Channels through CaMKII Activation. Lipids Health Dis. 19 (1), 189. 10.1186/s12944-020-01368-7 32825832 PMC7441649

[B68] Magierowska K. Korbut E. Hubalewska-Mazgaj M. Surmiak M. Chmura A. Bakalarz D. (2019). Oxidative Gastric Mucosal Damage Induced by Ischemia/reperfusion and the Mechanisms of its Prevention by Carbon Monoxide-Releasing Tricarbonyldichlororuthenium (II) Dimer. Free Radic. Biol. Med. 145, 198–208. 10.1016/j.freeradbiomed.2019.09.032 31568823

[B69] Männistö J. M. E. Maria M. Raivo J. Kuulasmaa T. Otonkoski T. Huopio H. (2020). Clinical and Genetic Characterization of 153 Patients with Persistent or Transient Congenital Hyperinsulinism. J. Clin. Endocrinol. Metab. 105 (4), dgz271. 10.1210/clinem/dgz271 32170320

[B70] Martin G. M. Sung M. W. Shyng S. L. (2020). Pharmacological Chaperones of ATP-Sensitive Potassium Channels: Mechanistic Insight from cryoEM Structures. Mol. Cell Endocrinol. 502, 110667. 10.1016/j.mce.2019.110667 31821855 PMC6994177

[B71] McClenaghan C. Huang Y. Yan Z. Harter T. M. Halabi C. M. Chalk R. (2020). Glibenclamide Reverses Cardiovascular Abnormalities of Cantu Syndrome Driven by KATP Channel Overactivity. J. Clin. Invest 130 (3), 1116–1121. 10.1172/JCI130571 31821173 PMC7269588

[B72] Moriguchi S. Ishizuka T. Yabuki Y. Shioda N. Sasaki Y. Tagashira H. (2018). Blockade of the KATP Channel Kir6.2 by Memantine Represents a Novel Mechanism Relevant to Alzheimer's Disease Therapy. Mol. Psychiatry 23 (2), 211–221. 10.1038/mp.2016.187 27777420

[B73] Mulder B. A. van Veldhuisen D. J. Rienstra M. (2021). Sudden Cardiac Death in Heart Failure: More Than Meets the Eye. Eur. J. Heart Fail 23 (8), 1361–1363. 10.1002/ejhf.2212 33932259

[B74] Noma A. (1983). ATP-regulated K+ Channels in Cardiac Muscle. Nature 305 (5930), 147–148. 10.1038/305147a0 6310409

[B75] Paggio A. Checchetto V. Campo A. Menabò R. Di Marco G. Di Lisa F. (2019). Identification of an ATP-Sensitive Potassium Channel in Mitochondria. Nature 572 (7771), 609–613. 10.1038/s41586-019-1498-3 31435016 PMC6726485

[B76] Papanicolaou K. N. Ashok D. Liu T. Bauer T. M. Sun J. Li Z. (2020). Global Knockout of ROMK Potassium Channel Worsens Cardiac Ischemia-Reperfusion Injury but Cardiomyocyte-specific Knockout Does Not: Implications for the Identity of mitoKATP. J. Mol. Cell Cardiol. 139, 176–189. 10.1016/j.yjmcc.2020.01.010 32004507 PMC7849919

[B77] Pereira O. Jr. Kowaltowski A. J. (2021). Mitochondrial K+ Transport: Modulation and Functional Consequences. Molecules 26 (10), 2935. 10.3390/molecules26102935 34069217 PMC8156104

[B78] Pertiwi K. R. Hillman R. M. Scott C. A. Chilton E. L. (2019). Ischemia Reperfusion Injury Produces, and Ischemic Preconditioning Prevents, Rat Cardiac Fibroblast Differentiation: Role of KATP Channels. J. Cardiovasc Dev. Dis. 6 (2), 22. 10.3390/jcdd6020022 PMC6617075 31167469

[B79] Pipatpolkai T. Usher S. Stansfeld P. J. Ashcroft F. M. (2020). New Insights into KATP Channel Gene Mutations and Neonatal Diabetes Mellitus. Nat. Rev. Endocrinol. 16 (7), 378–393. 10.1038/s41574-020-0351-y 32376986

[B80] Portal L. Morin D. Motterlini R. Ghaleh B. Pons S. (2019). The CO-releasing Molecule CORM-3 Protects Adult Cardiomyocytes against Hypoxia-Reoxygenation by Modulating pH Restoration. Eur. J. Pharmacol. 862, 172636. 10.1016/j.ejphar.2019.172636 31491405

[B81] Pui Ping C. Akhtar M. N. Israf D. A. Perimal E. K. Sulaiman M. R. (2020). Possible Participation of Ionotropic Glutamate Receptors and L-Arginine-Nitric Oxide-Cyclic Guanosine Monophosphate-ATP-Sensitive K+ Channel Pathway in the Antinociceptive Activity of Cardamonin in Acute Pain Animal Models. Molecules 25 (22), 5385. 10.3390/molecules25225385 PMC7698774 33217904

[B82] Rameshrad M. Omidkhoda S. F. Razavi B. M. Hosseinzadeh H. (2021). Evaluating the Possible Role of Mitochondrial ATP-Sensitive Potassium Channels in the Cardioprotective Effects of Morin in the Isolated Rat Heart. Life Sci. 264, 118659. 10.1016/j.lfs.2020.118659 33148418

[B83] Rieg A. D. Suleiman S. Bünting N. A. Verjans E. Spillner J. Schnöring H. (2020). Levosimendan Reduces Segmental Pulmonary Vascular Resistance in Isolated Perfused Rat Lungs and Relaxes Human Pulmonary Vessels. PLoS One 15 (5), e0233176. 10.1371/journal.pone.0233176 32421724 PMC7233573

[B84] Rosenfeld E. Ganguly A. De Leon D. D. (2019). Congenital Hyperinsulinism Disorders: Genetic and Clinical Characteristics. Am. J. Med. Genet. C Semin. Med. Genet. 181 (4), 682–692. 10.1002/ajmg.c.31737 31414570 PMC7229866

[B85] Rosenfeld E. Mitteer L. Boodhansingh K. Becker S. A. McKnight H. Boyajian L. (2021). Case Report: Two Distinct Focal Congenital Hyperinsulinism Lesions Resulting from Separate Genetic Events. Front. Pediatr. 9, 699129. 10.3389/fped.2021.699129 34336745 PMC8322518

[B86] Roubenne L. Marthan R. Le Grand B. Guibert C. (2021). Hydrogen Sulfide Metabolism and Pulmonary Hypertension. Cells 10 (6), 1477. 10.3390/cells10061477 34204699 PMC8231487

[B87] Sachse G. Haythorne E. Proks P. Stewart M. Cater H. Ellard S. (2020). Phenotype of a Transient Neonatal Diabetes Point Mutation (SUR1-R1183w) in Mice. Wellcome Open Res. 5, 15. 10.12688/wellcomeopenres.15529.2 34368464 PMC8323074

[B88] Sakamoto K. Kurokawa J. (2019). Involvement of Sex Hormonal Regulation of K+ Channels in Electrophysiological and Contractile Functions of Muscle Tissues. J. Pharmacol. Sci. 139 (4), 259–265. 10.1016/j.jphs.2019.02.009 30962088

[B89] Savarese G. Stolfo D. Sinagra G. Lund L. H. (2022). Heart Failure with Mid-range or Mildly Reduced Ejection Fraction. Nat. Rev. Cardiol. 19 (2), 100–116. 10.1038/s41569-021-00605-5 34489589 PMC8420965

[B90] Scala R. Maqoud F. Zizzo N. Mele A. Camerino G. M. Zito F. A. (2020). Pathophysiological Consequences of KATP Channel Overactivity and Pharmacological Response to Glibenclamide in Skeletal Muscle of a Murine Model of Cantù Syndrome. Front. Pharmacol. 11, 604885. 10.3389/fphar.2020.604885 33329006 PMC7734337

[B91] Severino P. D'Amato A. Netti L. Pucci M. Mariani M. V. Cimino S. (2020). Susceptibility to Ischaemic Heart Disease: Focusing on Genetic Variants for ATP-Sensitive Potassium Channel beyond Traditional Risk Factors. Eur. J. Prev. Cardiol. 1, 1. 10.1177/2047487320926780 33611546

[B92] Shin B. Saeed M. Y. Esch J. J. Guariento A. Blitzer D. Moskowitzova K. (2019). A Novel Biological Strategy for Myocardial Protection by Intracoronary Delivery of Mitochondria: Safety and Efficacy. JACC Basic Transl. Sci. 4 (8), 871–888. 10.1016/j.jacbts.2019.08.007 31909298 PMC6938990

[B93] Shoji T. Hayashi M. Sumi C. Kusunoki M. Uba T. Matsuo Y. (2019). Pharmacological Polysulfide Suppresses Glucose-Stimulated Insulin Secretion in an ATP-Sensitive Potassium Channel-dependent Manner. Sci. Rep. 9 (1), 19377. 10.1038/s41598-019-55848-7 31852936 PMC6920347

[B94] Sim J. H. Yang D. K. Kim Y. C. Park S. J. Kang T. M. So I. (2002). ATP-sensitive K(+) Channels Composed of Kir6.1 and SUR2B Subunits in guinea Pig Gastric Myocytes. Am. J. Physiol. Gastrointest. Liver Physiol. 282 (1), G137–G144. 10.1152/ajpgi.00057x.2002 11751167

[B95] Small H. Y. McNeilly S. Mary S. Sheikh A. M. Delles C. (2019). Resistin Mediates Sex-dependent Effects of Perivascular Adipose Tissue on Vascular Function in the Shrsp. Sci. Rep. 9 (1), 6897. 10.1038/s41598-019-43326-z 31053755 PMC6499830

[B96] Smeland M. F. McClenaghan C. Roessler H. I. Savelberg S. Hansen G. Å. M. Hjellnes H. (2019). ABCC9-related Intellectual Disability Myopathy Syndrome Is a KATP Channelopathy with Loss-Of-Function Mutations in ABCC9. Nat. Commun. 10 (1), 4457. 10.1038/s41467-019-12428-7 31575858 PMC6773855

[B97] Smith K. J. Neill M. Hoiland R. L. (2020). Scratching the Surface of Hypoxic Cerebral Vascular Control: a Potentially Polarizing View of Mechanistic Research in Humans. J. Physiol. 598 (16), 3313–3315. 10.1113/JP280244 32533733

[B98] Solhjoo S. O'Rourke B. (2015). Mitochondrial Instability during Regional Ischemia-Reperfusion Underlies Arrhythmias in Monolayers of Cardiomyocytes. J. Mol. Cell Cardiol. 78, 90–99. 10.1016/j.yjmcc.2014.09.024 25268650 PMC4268014

[B99] Sooklal C. R. López-Alonso J. P. Papp N. Kanelis V. (2018). Phosphorylation Alters the Residual Structure and Interactions of the Regulatory L1 Linker Connecting NBD1 to the Membrane-Bound Domain in SUR2B. Biochemistry 57 (44), 6278–6292. 10.1021/acs.biochem.8b00503 30273482

[B100] Stewart L. Turner N. A. (2021). Channelling the Force to Reprogram the Matrix: Mechanosensitive Ion Channels in Cardiac Fibroblasts. Cells 10 (5), 990. 10.3390/cells10050990 33922466 PMC8145896

[B101] Sudhir R. Du Q. Sukhodub A. Jovanović S. Jovanović A. (2020). Improved Adaptation to Physical Stress in Mice Overexpressing SUR2A Is Associated with Changes in the Pattern of Q-T Interval. Pflugers Arch. 472 (6), 683–691. 10.1007/s00424-020-02401-5 32458088 PMC7293680

[B102] Svalastoga P. Sulen Å. Fehn J. R. Aukland S. M. Irgens H. Sirnes E. (2020). Intellectual Disability in KATP Channel Neonatal Diabetes. Diabetes Care 43 (3), 526–533. 10.2337/dc19-1013 31932458

[B103] Takasawa K. Miyakawa Y. Saito Y. Adachi E. Shidei T. Sutani A. (2021). Marked Clinical Heterogeneity in Congenital Hyperinsulinism Due to a Novel Homozygous ABCC8 Mutation. Clin. Endocrinol. (Oxf) 94 (6), 940–948. 10.1111/cen.14443 33595839

[B104] Testai L. Sestito S. Martelli A. Gorica E. Flori L. Calderone V. (2021). Synthesis and Pharmacological Characterization of Mitochondrial KATP Channel Openers with Enhanced Mitochondriotropic Effects. Bioorg Chem. 107, 104572. 10.1016/j.bioorg.2020.104572 33418316

[B105] Udelson J. E. Lewis G. D. Shah S. J. Zile M. R. Redfield M. M. Burnett J. (2020). Effect of Praliciguat on Peak Rate of Oxygen Consumption in Patients with Heart Failure with Preserved Ejection Fraction: The CAPACITY HFpEF Randomized Clinical Trial. JAMA 324 (15), 1522–1531. 10.1001/jama.2020.16641 33079154 PMC7576408

[B106] Usher S. G. Ashcroft F. M. Puljung M. C. (2020). Nucleotide Inhibition of the Pancreatic ATP-Sensitive K+ Channel Explored with Patch-Clamp Fluorometry. Elife 9, e52775. 10.7554/eLife.52775 31909710 PMC7004565

[B107] Wang C. Cheng W. Bai S. Ye L. Du J. Zhong M. (2019). White Mulberry Fruit Polysaccharides Enhance Endothelial Nitric Oxide Production to Relax Arteries *In Vitro* and Reduce Blood Pressure *In Vivo* . Biomed. Pharmacother. 116, 109022. 10.1016/j.biopha.2019.109022 31154271

[B108] Wang C. Liu L. Wang Y. Xu D. (2021). Advances in the Mechanism and Treatment of Mitochondrial Quality Control Involved in Myocardial Infarction. J. Cell Mol. Med. 25 (15), 7110–7121. 10.1111/jcmm.16744 34160885 PMC8335700

[B109] Wang S. Guo X. Long C. L. Li C. Zhang Y. F. Wang J. (2019). SUR2B/Kir6.1 Channel Openers Correct Endothelial Dysfunction in Chronic Heart Failure via the miR-1-3p/ET-1 Pathway. Biomed. Pharmacother. 110, 431–439. 10.1016/j.biopha.2018.11.135 30530045

[B110] Wang W. L. Ge T. Y. Chen X. Mao Y. Zhu Y. Z. (2020). Advances in the Protective Mechanism of NO, H2S, and H2 in Myocardial Ischemic Injury. Front. Cardiovasc Med. 7, 588206. 10.3389/fcvm.2020.588206 33195476 PMC7661694

[B111] White D. S. Chowdhury S. Idikuda V. Zhang R. Retterer S. T. Goldsmith R. H. (2021). cAMP Binding to Closed Pacemaker Ion Channels Is Non-cooperative. Nature 595 (7868), 606–610. 10.1038/s41586-021-03686-x 34194042 PMC8513821

[B112] Yang H. Q. Jana K. Rindler M. J. Coetzee W. A. (2018). The Trafficking Protein, EHD2, Positively Regulates Cardiac Sarcolemmal KATP Channel Surface Expression: Role in Cardioprotection. FASEB J. 32 (3), 1613–1625. 10.1096/fj.201700027R 29133341 PMC5892718

[B113] Yang H. Q. Martinez-Ortiz W. Hwang J. Fan X. Cardozo T. J. Coetzee W. A. (2020). Palmitoylation of the KATP Channel Kir6.2 Subunit Promotes Channel Opening by Regulating PIP2 Sensitivity. Proc. Natl. Acad. Sci. U. S. A. 117 (19), 10593–10602. 10.1073/pnas.1918088117 32332165 PMC7229695

[B114] Yang L. Li R. C. Xiang B. Li Y. C. Wang L. P. Guo Y. B. (2021). Transcriptional Regulation of Intermolecular Ca2+ Signaling in Hibernating Ground Squirrel Cardiomyocytes: The Myocardin-Junctophilin axis. Proc. Natl. Acad. Sci. U. S. A. 118 (14), e2025333118. 10.1073/pnas.2025333118 33785600 PMC8040632

[B115] Yang M. Dart C. Kamishima T. Quayle J. M. (2020). Hypoxia and Metabolic Inhibitors Alter the Intracellular ATP:ADP Ratio and Membrane Potential in Human Coronary Artery Smooth Muscle Cells. PeerJ 8, e10344. 10.7717/peerj.10344 33240653 PMC7664465

[B116] Yu H. Zhang F. Yan P. Zhang S. Lou Y. Geng Z. (2021). LARP7 Protects against Heart Failure by Enhancing Mitochondrial Biogenesis. Circulation 143 (20), 2007–2022. 10.1161/CIRCULATIONAHA.120.050812 33663221

[B117] Yu L. Li W. Park B. M. Lee G.-J. Kim S. H. (2019). Hypoxia Augments NaHS-Induced ANP Secretion via KATP Channel, HIF-1α and PPAR-γ Pathway. Peptides 121, 170123. 10.1016/j.peptides.2019.170123 31386893

[B118] Zhang D. M. Lin Y. F. (2020). Functional Modulation of Sarcolemmal KATP Channels by Atrial Natriuretic Peptide-Elicited Intracellular Signaling in Adult Rabbit Ventricular Cardiomyocytes. Am. J. Physiol. Cell Physiol. 319 (1), C194–C207. 10.1152/ajpcell.00409.2019 32432931 PMC7468895

[B119] Zhang H. Hanson A. de Almeida T. S. Emfinger C. McClenaghan C. Harter T. (2021). Complex Consequences of Cantu Syndrome SUR2 Variant R1154Q in Genetically Modified Mice. JCI Insight 6 (5), e145934. 10.1172/jci.insight.145934 PMC8021106 33529173

[B120] Zhang H. X. Silva J. R. Lin Y. W. Verbsky J. W. Lee U. S. Kanter E. M. (2013). Heterogeneity and Function of K(ATP) Channels in Canine Hearts. Heart rhythm. 10 (10), 1576–1583. 10.1016/j.hrthm.2013.07.020 23871704 PMC3816016

[B121] Zhang X. Zhang X. Xiong Y. Xu C. Liu X. Lin J. (2016). Sarcolemmal ATP-Sensitive Potassium Channel Protects Cardiac Myocytes against Lipopolysaccharide-Induced Apoptosis. Int. J. Mol. Med. 38 (3), 758–766. 10.3892/ijmm.2016.2664 27430376 PMC4990318

[B122] Zhang Y. Wang X. Liu R. Li Q. Tian W. Lei H. (2021). The Effectiveness and Safety of Nicorandil in the Treatment of Patients with Microvascular Angina: A Protocol for Systematic Review and Meta-Analysis. Med. Baltim. 100 (2), e23888. 10.1097/MD.0000000000023888 PMC7808505 33466132

[B123] Zhao G. Kaplan A. Greiser M. Lederer W. J. (2020). The Surprising Complexity of KATP Channel Biology and of Genetic Diseases. J. Clin. Invest 130 (3), 1112–1115. 10.1172/JCI135759 32065592 PMC7269573

[B124] Zhao Y. Ge J. Li X. Guo Q. Zhu Y. Song J. (2019). Vasodilatory Effect of Formaldehyde via the NO/cGMP Pathway and the Regulation of Expression of KATP, BKCa and L-type Ca^2+^ Channels. Toxicol. Lett. 312, 55–64. 10.1016/j.toxlet.2019.04.006 30974163 PMC7790163

[B125] Zhou M. Yoshikawa K. Akashi H. Miura M. Suzuki R. Li T. S. (2019). Localization of ATP-Sensitive K+ Channel Subunits in Rat Liver. World J. Exp. Med. 9 (2), 14–31. 10.5493/wjem.v9.i2.14 31938690 PMC6955576

[B126] Zhou Z. Y. Zhao W. R. Shi W. T. Xiao Y. Ma Z. L. Xue J. G. (2019). Endothelial-Dependent and Independent Vascular Relaxation Effect of Tetrahydropalmatine on Rat Aorta. Front. Pharmacol. 10, 336. 10.3389/fphar.2019.00336 31057398 PMC6477965

[B127] Zi C. Zhang C. Yang Y. Ma J. (2020). Penehyclidine Hydrochloride Protects against Anoxia/reoxygenation Injury in Cardiomyocytes through ATP-Sensitive Potassium Channels, and the Akt/GSK-3β and Akt/mTOR Signaling Pathways. Cell Biol. Int. 44 (6), 1353–1362. 10.1002/cbin.11329 32125033

